# Photonic thermal management of overhead cables via high-thermal-conductivity radiative cooling

**DOI:** 10.1038/s41377-026-02485-1

**Published:** 2026-10-08

**Authors:** Kai Xu, Peng-Fei Cao, Jehad K. El-Demellawi, Hussam Qasem, Qiaoqiang Gan

**Affiliations:** 1 Division of Physical Science and Engineering, King Abdullah University of Science and Technology (KAUST), Thuwal, Saudi Arabia; 2 Center for Renewable Energy and Storage Technologies (CREST), King Abdullah University of Science and Technology (KAUST), Thuwal, Saudi Arabia; 3 State Key Laboratory of Organic-Inorganic Composites, College of Materials Science and Engineering, Beijing University of Chemical Technology, Beijing, China; 4 Future Energy Technology Institute, King Abdulaziz City for Science and Technology, Riyadh, Saudi Arabia

**Keywords:** Polymers, Photonic devices

## Abstract

As the thermal backbone of urban energy infrastructure, transmission cables directly affect the stability and resilience of modern cities. However, high operating temperatures, driven by Joule heating and solar radiation, critically limit the ampacity of overhead transmission lines, particularly under extreme climate conditions. Such high temperatures constrain electricity delivery and pose systemic risks to essential services and long-term urban sustainability. To overcome this challenge, we adopt a high thermal conductivity radiative cooling (HTCRC) coating comprising hexagonal boron nitride (h-BN) nanoparticles embedded in a polydimethylsiloxane matrix to reduce cable temperature. Enabled by photonic thermal management, this composite structure simultaneously achieves high solar reflectance (0.92) through efficient light scattering, strong mid-infrared emissivity via polymer vibrational modes (0.95), and enhanced thermal conductivity (1.28 W m⁻¹ K⁻¹). Outdoor experiments demonstrate notable daytime cooling of simulated HTCRC-coated power cables, maintaining safe operating temperatures even under intense solar exposure. In addition, thermal modeling based on the CIGRE 601 standard reveals a 6–10% increase in ampacity across representative hot-climate cities globally. These findings highlight high thermal conductivity radiative cooling as an effective and scalable strategy to enhance urban power grid performance, improve energy delivery security, and support the development of climate-resilient cities under rapidly growing electricity demand driven by electrification and the expanding energy footprint of artificial intelligence.

**Figure:**
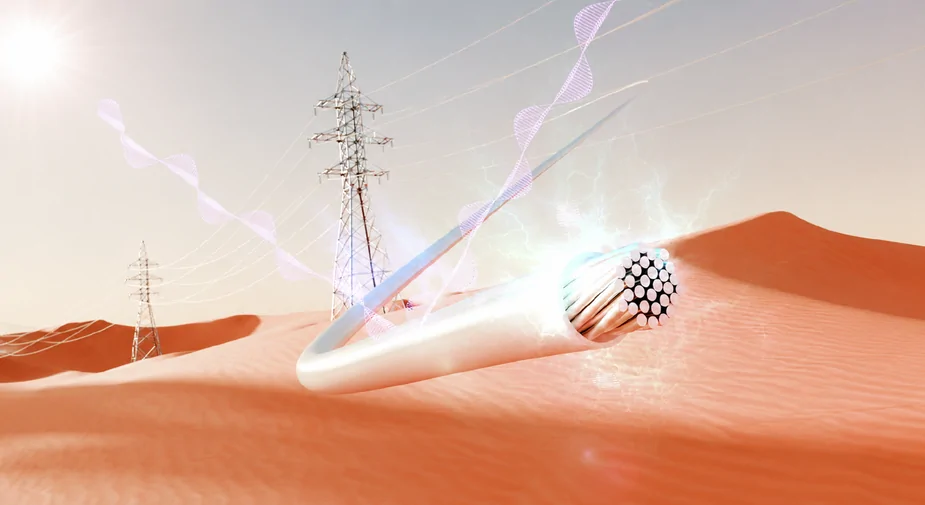

## Introduction

The electrical grid functions as the circulatory system of modern cities, sustaining economic productivity, social stability, and the continuous operation of essential urban services^1^. As cities intensify and electrify, this critical infrastructure is facing unprecedented strain^2^. Driven by transport electrification, expanding data centers, and the increasing energy intensity of artificial intelligence, the rapid increase in electricity demand coincides with accelerating urbanization and warming climates^3–5^. The International Energy Agency projects that global electricity consumption will approximately double by 2050^6^, placing mounting thermal stress on the overhead transmission lines that form the backbone of power delivery in cities worldwide. Increasingly, the thermal limits of existing transmission infrastructure are constraining grid capacity, emerging as a critical bottleneck for reliable power supply and the integration of renewable energy into urban systems^7^, especially under rapidly growing electricity demand driven by electrification and the expanding energy footprint of artificial intelligence^8^.

These infrastructural pressures are further amplified by the increasing frequency and severity of heatwaves^9–12^. Extreme heat events disproportionately affect cities, where elevated ambient temperatures and reduced convective cooling exacerbate thermal loading on overhead conductors. In many regions (particularly across the Global South), overhead lines remain the most prevalent and cost-effective transmission architecture, rendering urban power networks especially vulnerable to climate-driven thermal stress. Collectively, increasing demand and intensifying heat highlight an urgent need to enhance the thermal resilience of urban transmission infrastructure to ensure reliable electricity delivery in a warming world.

A fundamental physical constraint lies at the core of this challenge. Overhead conductors, typically aluminum conductor steel-reinforced (ACSR) cables, are limited by the balance between internal resistive (Joule) heating and passive heat dissipation, governing their maximum current-carrying capacity (ampacity)^13^. This limit is defined by a maximum allowable operating temperature (typically ~75 °C for ACSR conductors^14,15^), which is routinely approached or exceeded under extreme ambient conditions. In hot urban climates, where air temperatures can surpass 50 °C^16,17^, the diminished thermal gradient between the conductor and the environment severely degrades convective and radiative cooling. The consequences for cities are substantial, e.g., increased resistive losses owing to elevated conductor temperatures^18^, accelerated sag^19^, induced mechanical degradation through annealing^13^, and increased risks of wildfires and cascading blackouts, which can disrupt essential urban services^20,21^. A 10 °C increase in conductor temperature can increase the electrical resistance of aluminum conductors and the associated Joule-heating losses by approximately 4%, resulting in additional energy losses during power transmission^13^. From an economic and reliability perspective, heat-related grid stress can be substantial. For example, recent outage data from China show that heatwaves increase outage frequency by 3.9–4.0% and outage duration by 7.9–8.3%, while each 1 °C increase in temperature raises outages by approximately 0.1%^21^. The associated economic losses of heatwave-induced outages were estimated to reach approximately US$3.4 billion in the analyzed scenario.

Existing mitigation strategies offer only partial relief. For example, dynamic line rating systems^22^ improve the utilization of existing thermal margins but are fundamentally reactive because they do not directly reduce conductor temperature. In addition, high-temperature low-sag conductors^23^ can tolerate higher operating temperatures but require extensive retrofitting of transmission infrastructure, which limits their economic feasibility for widespread urban deployment. Thus, enhancing passive cooling through surface engineering of existing conductors emerges as a broadly applicable approach. However, conventional conductor surfaces and insulating coatings are optically and thermally suboptimal; e.g., aged bare aluminum cable exhibits modest solar reflectance and mid-infrared emissivity^24^, and common polymeric insulators absorb substantial solar radiation^25^ and possess low thermal conductivity^26^, thereby trapping internally generated heat and undermining surface-based cooling strategies.

In this context, an ideal cable surface should exhibit high solar reflectance to suppress absorption and strong mid-infrared (MIR) emissivity to enhance radiative heat loss. While the concept of radiative cooling has been successfully established^27^ and such materials have found broad applications in areas including building insulation and smart textiles^28–32^, their low thermal conductivity renders them ineffective for systems with internal heat sources like power cables, where efficient heat transfer from the conductor core to the surface is essential^33^. Therefore, there is a clear need for radiative cooling coatings that combine optical selectivity with high thermal conductivity to enable efficient extraction of internally generated heat while maintaining radiative cooling capability for overhead cable applications.

To address these challenges, we introduced a high thermal conductivity radiative cooling (HTCRC) coating engineered to enhance the thermal resilience of overhead transmission lines in extreme urban environments. Unlike conventional radiative cooling coatings primarily designed for passive cooling of static planar surfaces, the HTCRC coating is specifically developed for internally heated cylindrical conductors, where efficient extraction of Joule heat requires simultaneous optimization of solar reflection, infrared emission, thermal conductivity, and critical-radius effects. The proposed coating is a polymer–ceramic composite formed by dispersing hexagonal boron nitride (h-BN) particles within a polydimethylsiloxane (PDMS) matrix that achieves a solar reflectance of 0.92, a mid-infrared emissivity of 0.95, and an effective thermal conductivity of 1.28 W m^−^¹ K^−^¹. Beyond optical selectivity, the cylindrical coating is designed to satisfy the critical insulation radius condition for overhead cables, which ensures that it does not introduce an additional thermal barrier. Instead, the HTCRC layer reduces the total thermal resistance of the conductor–environment system and, therefore, yields measurable decreases in the steady-state operating temperature and cumulative Joule losses when integrated over long-span overhead lines under sustained load. The combined effects of the suppressed solar heat gain and enhanced dissipation of internally generated heat are particularly effective under the peak-load, low-wind conditions observed during stagnant urban heatwaves. Notably, in the present work, radiative cooling refers specifically to enhanced infrared radiative heat rejection from the cable surface, while the overall temperature reduction arises from the combined effects of suppressed solar heat gain, radiative heat dissipation, and efficient transport of internally generated Joule heat. Field-relevant simulations for Riyadh, Saudi Arabia, demonstrate that the proposed HTCRC coatings can increase conductor ampacity by up to 18.3% under extreme summer conditions (45 °C ambient temperature). By passively improving the current-carrying capacity, stability, and safety, this scalable approach offers a practical pathway to improve the resilience of urban power networks in a warming climate.

## Results

### Design rationale for HTCRC coating

The ampacity of an overhead conductor is defined as the maximum allowable current that can be carried without exceeding its prescribed thermal limit^13^. This limit is fundamentally determined by the balance between internally generated Joule heating and heat dissipation to the surrounding environment. Under high electrical loading and elevated ambient temperature conditions, weakened convective and radiative heat transfer increasingly drive conductor temperatures toward the thermal limit of ACSR cables. Field measurements on operating overhead lines corroborate this behavior, with conductor temperatures observed to approach ~73 °C under high load currents (~700 A) and peak solar irradiance exceeding 1200 W m^−2^^34^. Elevated operating temperatures simultaneously exacerbate resistive losses, accelerate mechanical sag, reduce ground clearance, and undermine long-term operational reliability.

To overcome these vulnerabilities, the proposed HTCRC coating integrates three synergistic thermal management functions, i.e., high solar reflectance to suppress solar absorption, strong mid-infrared emissivity to maximize passive radiative cooling, and enhanced thermal conductivity to facilitate rapid transport of Joule heat to the outer surface of the coated cable. The cylindrical coating design also maintains the effective insulation radius within acceptable limits, which enables seamless retrofitting onto existing conductor geometries without compromising electrical clearance or corona performance. By combining thermal and optical functionalities within this scalable polymer–ceramic composite, the proposed HTCRC coating directly addresses the intrinsic thermal bottleneck of overhead lines.

### Thermal design: critical insulation radius of HTCRC coating

**Fig. 1:**
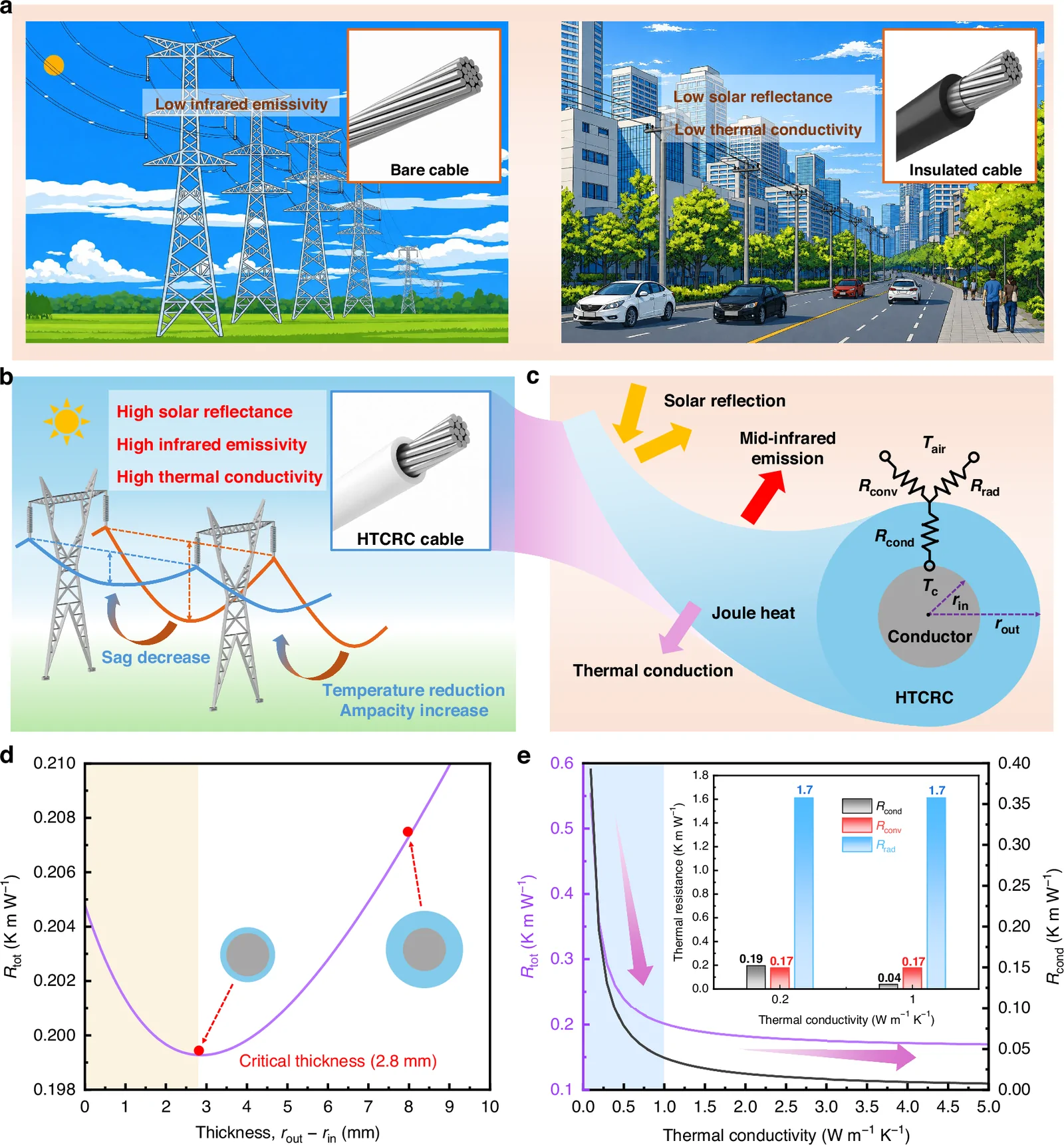
Thermal design framework of proposed HTCRC coating for overhead power cables. **a** (left) Conventional bare overhead cable used in long-span transmission lines; (right) polymer-insulated overhead cable in urban environments. **b** Conceptual strategy for proposed HTCRC coating: applied to bare or insulated cables to reduce sag, lower temperature, and enhance ampacity under solar exposure. **c** Schematic of the design rationale (high solar reflectance, high mid-infrared emissivity, and high thermal conductivity) and thermal resistance model for HTCRC-coated cables, showing the series-parallel combination of conductive ${R}_{{{\rm{cond}}}}$, convective ${R}_{{{\rm{conv}}}}$, and radiative ${R}_{{{\rm{rad}}}}$ components. **d** Calculated total thermal resistance ${R}_{{{\rm{tot}}}}$ as a function of coating thickness. **e** Influence of thermal conductivity on conductive and total resistance. The inset compares the relative contributions of the conductive, convective, and radiative thermal resistances at low and high thermal conductivities

In classical thermal management of insulated fluid pipelines, the critical insulation radius *r*_cr_ is a key design parameter governing the transition between enhanced and suppressed heat loss^35^. Owing to the cylindrical geometry, the total thermal resistance of a pipeline is reduced with increasing insulation thickness until *r*_cr_ is reached, beyond which additional insulation increases thermal resistance (Note S1). For overhead conductors, the design objective is fundamentally inverted. Here, rather than suppressing heat transfer, thermal performance can be optimized by approaching the critical insulation radius, at which dissipation of the internally generated Joule heat is maximized. This counterintuitive principle repurposes a concept conventionally used for thermal insulation into a mechanism to actively enhance heat rejection from power cables. In practice, bare aluminum conductors are favored in high-voltage transmission to minimize dielectric losses and simplify heat dissipation, whereas polymer-insulated cables are required in medium-voltage urban distribution for safety in densely built environments^36^. Figure 1a illustrates the two prevailing conductor paradigms, i.e., bare aluminum cables used in high-voltage transmission (left) and polymer-insulated cables used in medium-voltage urban distribution (right). Bare conductors inherently exhibit weak radiative cooling, whereas conventional polymer insulators (e.g., cross-linked polyethylene (XLPE), κ ≈ 0.3 W m^−1^ K^−1^^26^) provide electrical safety but severely hinder heat dissipation. Consequently, when exposed to intense solar irradiation, both types of cables can experience excessive temperature rise, which accelerates resistive losses, increases sag, and compromises operational reliability.

To address these limitations, we propose the HTCRC coating as the outermost layer to reduce cable temperature, enhance ampacity, and mitigate sag development (Fig. 1b). To quantitatively evaluate the heat dissipation enhancement of this strategy, we first constructed a thermal resistance network model (Fig. 1c) that represents the conductor, HTCRC coating, and surrounding environment as a coupled series-parallel pathway involving conduction, convection, and radiation. The total thermal resistance *R*_tot_ can be expressed as follows:

1 $${R}_{{{\rm{tot}}}}={R}_{{{\rm{cond}}}}+\frac{1}{\frac{1}{{R}_{{{\rm{conv}}}}}+\frac{1}{{R}_{{{\rm{rad}}}}}}$$

where${R}_{{{\rm{cond}}}}$,${R}_{{{\rm{conv}}}}$, and ${R}_{{{\rm{rad}}}}$ denote the conductive, convective, and radiative thermal resistances, respectively (refer to Note S1 for additional details). Herein, a typical cable radius of 10 mm was employed for a representative analysis.

Figure 1d shows that for coatings with κ ≈ 1.0 W m^−1^ K^−1^, the total thermal resistance initially decreases with increasing thickness and reaches a minimum at a critical thickness of 2.8 mm, corresponding to an overall reduction of ~3% relative to the uncoated conductor. Within this regime, the additional coating paradoxically enhances heat dissipation by increasing the effective surface area available for convective and radiative heat transfer. However, beyond the critical thickness, the added radial conduction path becomes dominant, which leads to an increase in total thermal resistance and a corresponding reduction in cooling efficiency (Fig. S1). In practical cable systems, the HTCRC coating is intended to serve as an outer thermal-management layer that complements existing electrical insulation and mechanical protection layers. Therefore, the coating thickness should be optimized according to conductor radius, existing insulation thickness, voltage level, thermal conductivity, and local environmental conditions to maintain the total cable radius near the favorable critical-radius regime. Intriguingly, the critical thickness is influenced by cable radius and wind speed because both determine the convective heat transfer coefficient (Fig. S2). Larger cable radii and stronger convective heat transfer generally reduce the critical thickness, whereas smaller conductors or weaker convection shift the critical thickness toward larger values. Thus, in practice, the optimal coating thickness should be determined by conductor geometry and the local climate. For example, wind speeds of 4–8 m s^−1^ at an elevation of 100 m in Riyadh^37^ shift the optimal range but remain compatible with International Electrotechnical Commission (IEC) 60502 standards for insulation thickness^38^. Thus, coatings designed for this environmental condition can provide dielectric safety and net cooling benefits.

The thermal conductivity of the HTCRC coating critically governs the overall heat dissipation capability of the cable system. As the sole pathway for transferring internally generated Joule heat from the aluminum core to the outer surface, it directly influences the conductive component of the total thermal resistance network. For example, increasing κ from 0.2 to ~1 W m^−1^ K^−1^ dramatically reduces *R*_tot_, which highlights the importance of efficient Joule heat transport to the surface of the cable (Fig. 1e). Above 1 W m^−1^ K^−1^, diminishing returns appear because convection and radiation become the limiting factors (Fig. 1e, inset; see Note S2 for the heat-flux decomposition analysis). In summary, this thermal analysis defines one side of the practical design target for next-generation overhead transmission systems, i.e., coatings with κ ≈ 1.0 W m^−1^ K^−1^ that balance insulation, radiative cooling, and conductive heat spreading offer a lightweight and effective thermal management strategy. Complementary to thermal transport considerations, the optical design of the cable coating is equally critical.

### Optical design and manufacturing of HTCRC coating

Most reported RC coatings were developed for building envelopes, where low thermal conductivity is advantageous in terms of suppressing conductive heat gain^27,30,32,39^. Recent analyses have also emphasized the inherent tradeoff between radiative performance and thermal transport in these systems^40,41^. Distinct from these applications, overhead conductors require a fundamentally different coating paradigm that simultaneously delivers high solar reflectance, strong mid-infrared emissivity, and, critically, high thermal conductivity to extract the internally generated Joule heat from the core of the cable. To satisfy these coupled optical and thermal requirements, h-BN, which uniquely combines strong optical scattering, high dielectric contrast, and ultrahigh intrinsic thermal conductivity (>400 W m^−1^ K^−1^)^42^, was selected as the scattering medium in this study. This choice enables the coating to simultaneously suppress solar heat absorption and accelerate phonon-mediated heat transport. The polymer matrix, i.e., PDMS, contributes to strong vibrational absorption and re-emission in the mid-infrared regime, thereby ensuring high emissivity for effective radiative cooling. Collectively, the h-BN/PDMS composite transforms conventional low-conductivity RC films into dual-functional cooling layers tailored for heat-generating overhead cables.

**Fig. 2:**
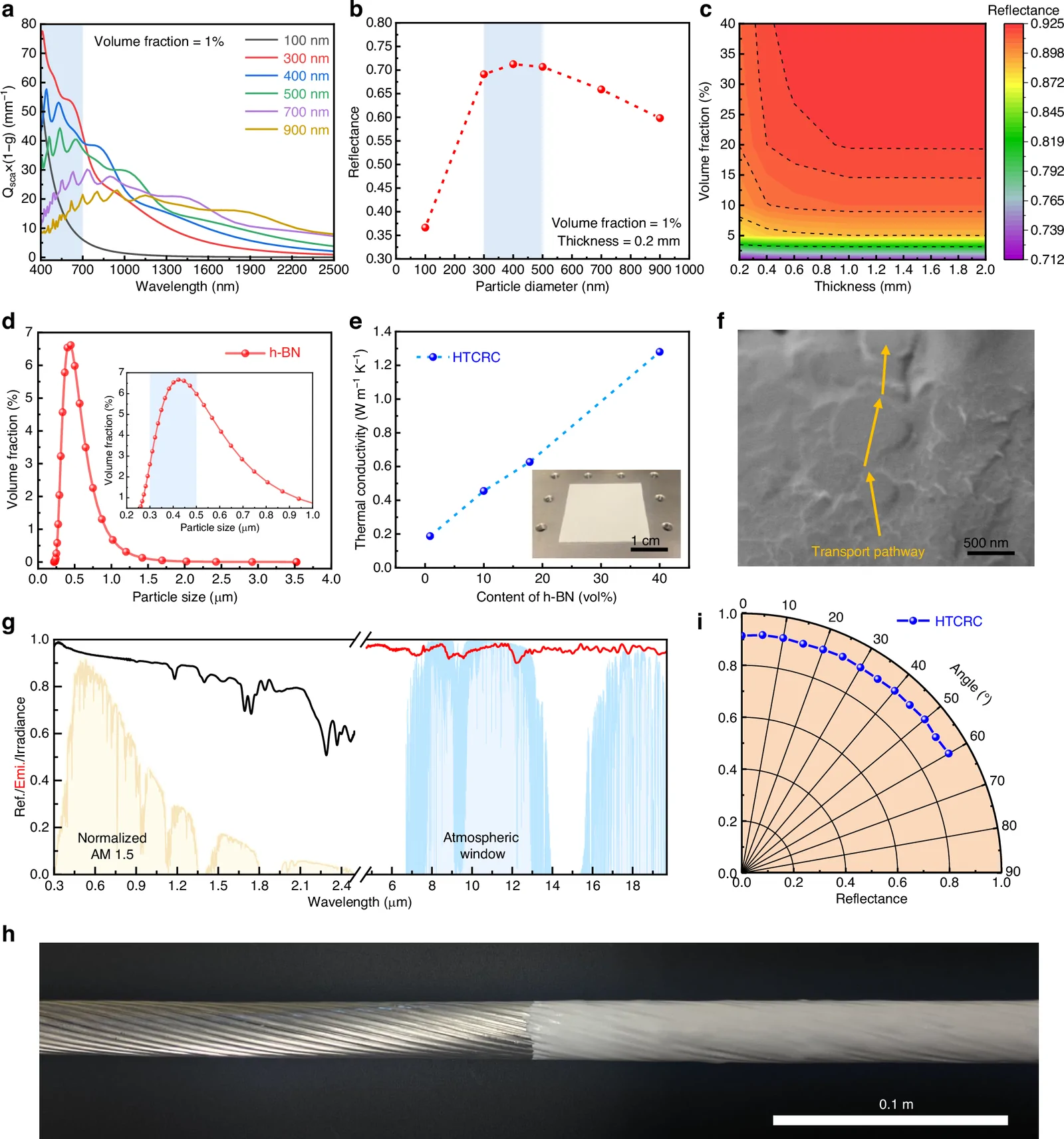
Optical and thermal characterization of HTCRC coating. **a** Calculated backscattering performance of h-BN particles as a function of particle size, indicating the optimal size range (300 − 500 nm) for solar spectrum reflection. **b** Simulated reflectance of HTCRC coating using Monte Carlo ray tracing. **c** Simulated total solar reflectance as a function of particle volume fraction and coating thickness. **d** Particle size distribution of h-BN showing majority of particles within the optimal scattering size range. **e** Measured thermal conductivity of the HTCRC coating as a function of h-BN volume fraction (the inset shows the fabricated HTCRC coating). **f** Cross-sectional SEM image of the HTCRC coating. **g** Solar reflectance and mid-infrared emissivity spectra of the coating, overlaid with AM1.5 solar spectrum and atmospheric window. **h** Bare cable before and after applying the HTCRC coating. **i** Angular-dependent solar reflectance of the HTCRC coating, confirming weak angle dependence and stable performance throughout the day

Building on the aforementioned thermal conductivity target of 1 W m^−1^ K^−1^ set by the thermal design, we optimized the optical properties of the coating. As shown in Fig. 2a, a Lorenz–Mie scattering model reveals that solar backscattering is maximized for h-BN particle diameters of 300–500 nm, coinciding with the peak energy of the solar spectrum (Notes S3 and S4). Furthermore, Monte Carlo ray tracing simulations (Fig. 2b) confirmed that coatings incorporating 300–500-nm particles yielded the highest solar reflectance (Notes S5 and S6). In addition, this solar reflectance increases with the h-BN volume fraction up to ~10%, beyond which additional loading yields diminishing gains (Fig. 2c). As shown in Fig. 2c, the coating thickness exhibited weak influence, with reflectance saturating near 0.91 for thicknesses of 0.2–2 mm. These findings define the target design window, i.e., particle size distribution centered at 400 nm, ~10% volume fraction, and coating thickness that operates near the critical insulation radius, where the total thermal resistance is minimized.

Following this target, we fabricated the HTCRC coating by dispersing h-BN nanoplatelets into PDMS. As demonstrated by the results of the particle size distribution analysis in Fig. 2d, most platelets used in our experiment fall within the optimal 300–500 nm scattering range. As a result, the thermal conductivity of the PDMS film increased from 0.18 W m^−1^ K^−1^ for pure PDMS to 1.28 W m^−1^ K^−1^ at 40% h-BN loading (Fig. 2e). Specifically, the cross-sectional SEM image (Fig. 2f) shows that the HTCRC coating at this concentration exhibits a percolated h-BN network, which creates efficient phonon transport pathways, overcomes interfacial resistance, and enables long-range heat conduction. This conductivity surpasses the threshold required to render conduction resistance nonlimiting in overhead cable cooling, which validates the proposed thermal design principle. In addition, at a h-BN volume fraction of 40%, the bulk density of the HTCRC coating is ~1.2 g cm^−3^, which is well within the typical density range of commercial cable insulation materials (~0.9–1.4 g cm^−3^^43^). This indicates that applying the coating at thicknesses that are comparable to those of commercial cable insulation layers (i.e., 0–8 mm, from the IEC 60502 standard) does not introduce additional weight or tensile loading and therefore does not compromise the conductor’s mechanical integrity.

Remarkably, the optical feature of the fabricated HTCRC coating is also compelling (Fig. 2g), i.e., the solar reflectance reaches 0.92 with reflectance extending into the ultraviolet, providing both enhanced solar rejection and photostability, while the mid-infrared emissivity reaches 0.95. Figure 2h compares the appearance of the bare cable before and after applying the HTCRC coating. Notably, because the coating is applied onto a commercial cylindrical cable, achieving effective rejection of nominally parallel solar irradiation requires the HTCRC coating to maintain high reflectance over a broad range of incidence angles. The results of angular-resolved measurements further confirm minimal dependence of solar reflectance on incidence angle (Fig. 2i), which ensures stable optical performance under varying solar illumination conditions. These results demonstrate that dispersing h-BN into a PDMS matrix yields a retrofittable coating with dual capability, realizing both high thermal conductivity and strong radiative cooling. This integrated functionality is absent in conventional RC films and directly addresses the thermal management bottleneck inherent in overhead cables (see Note S7 for a comparison between the HTCRC coating and representative radiative cooling materials). By uniting optical scattering, thermal conduction, and infrared emission within a scalable composite, the proposed HTCRC coating establishes a new paradigm for RC materials tailored to energy infrastructure. However, this paradigm shift may also introduce new practical challenges because mechanical reliability and long-term durability must be analyzed carefully and optimized for long-term operation in outdoor environments.

### Mechanical reliability and environmental durability

**Fig. 3:**
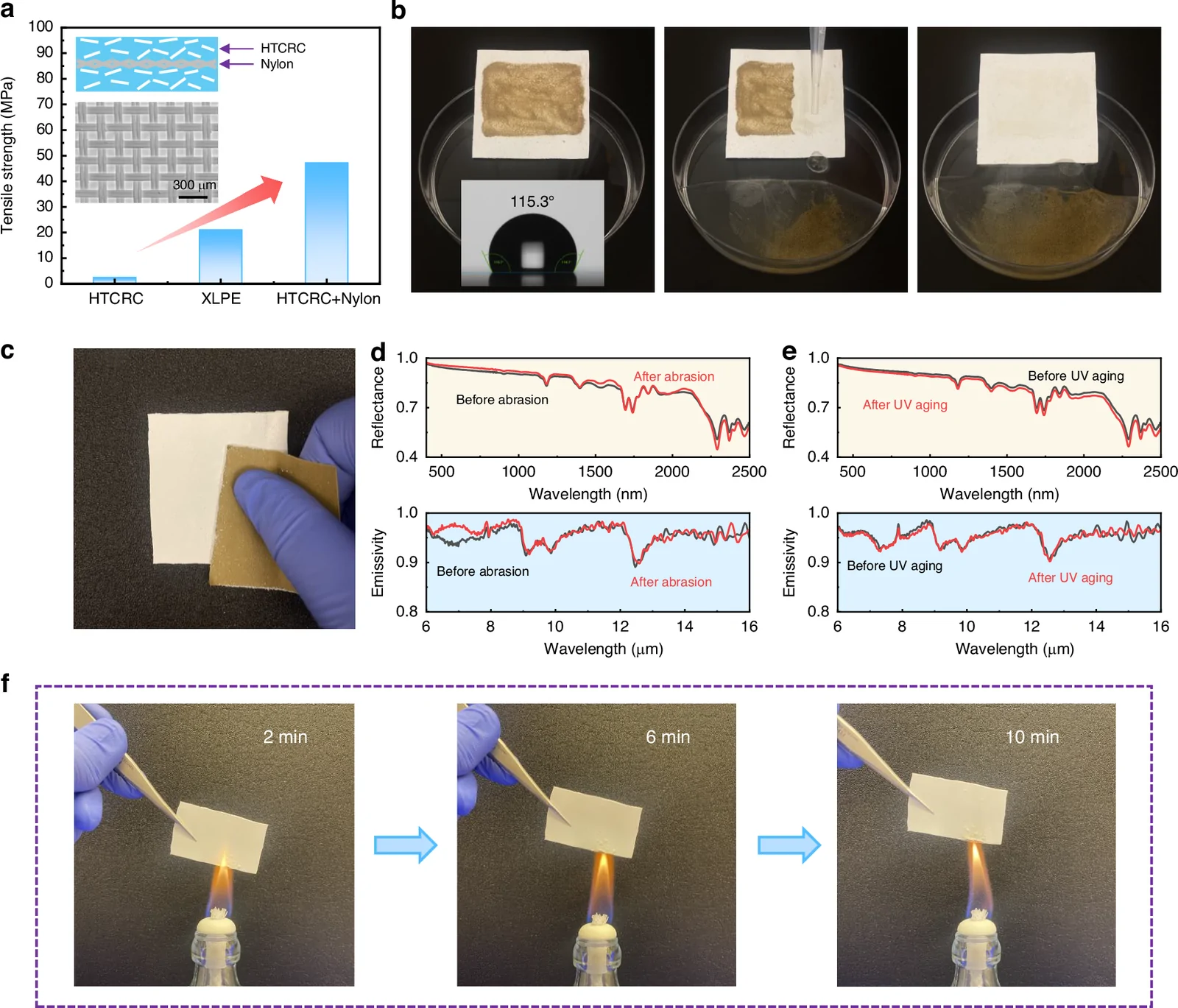
Mechanical robustness and environmental durability of proposed HTCRC coating. **a** Tensile strength of pristine HTCRC, XLPE, and Nylon-enhanced HTCRC. The inset shows a structural schematic of the Nylon-enhanced HTCRC and the microscopic morphology of the pores on the Nylon layer. **b** Dust-resistant performance of proposed HTCRC coating. The inset shows the contact angle on the HTCRC surface, confirming its hydrophobic dust-resistant property. **c** Sandpaper abrasion test conducted to simulate windblown particle erosion. **d** Optical performance before and after abrasion. **e** Optical performance before and after UV-accelerated aging. **f** Fire resistance test showing structural integrity under exposure to direct flame

Considering the harsh environmental conditions experienced by overhead cables (especially in tropical regions), the HTCRC coating must possess sufficient mechanical robustness and long-term durability to ensure safe and stable operation under prolonged exposure to extreme stressors. During cable installation and long-term operation, tensile forces are routinely exerted on the surface of the cable; thus, the coating must have a high level of mechanical robustness. The results of tensile tests (Fig. 3a) demonstrate that the intrinsic tensile strength of a 2 mm-thick HTCRC coating is 2.5 MPa, which is less than that of a 2-mm-thick XLPE film (21 MPa), a common material used for cable insulation layers. This mechanical limitation can be easily addressed by introducing an intermediate 0.13-mm-thick Nylon layer into the 2-mm-thick HTCRC film (refer to the inset in Fig. 3a and the fabrication details in Note S8). Therefore, the tensile strength of the Nylon-supported HTCRC film can be improved to ~45 MPa, which is more than twice that of the XLPE film; thus, it satisfies the mechanical requirements of cable insulation layers according to ASTM D1248^44^. Notably, the thermal conductivity of the Nylon-enhanced HTCRC remains ~1.2 W m^−1^ K^−1^, which indicates that incorporating the Nylon layer has a negligible impact on the high thermal conductivity of the coating because the Nylon layer is very thin (~0.15 mm) and possesses a porous structure (Fig. 3a, inset), allowing the HTCRC matrix to thoroughly infiltrate the pores and form continuous heat transfer pathways (Note S9).

Intriguingly, the HTCRC coating also exhibits intrinsic dust-resistant capability against dust accumulation. Figure 3b shows its hydrophobic surface feature (with a contact angle of 115.3°), allowing recovery of the original white appearance after cleaning the dust layer. This feature is particularly beneficial in dust-prone environments (Note S10). In addition, in arid regions, e.g., Saudi Arabia, sandstorms frequently subject outdoor cables to abrasive forces, making resistance to windblown sand erosion an additional critical requirement. We simulated this condition using standardized sandpaper abrasion tests (Note S11). As shown in Fig. 3c, the surface of the coating retains its white appearance even after 1000-cycle repeated polishing. Corresponding spectral measurements further confirm that the solar and infrared optical properties of the HTCRC coating remain essentially unchanged after abrasion (Fig. 3d), demonstrating excellent resistance to mechanical wear.

To further demonstrate the HTCRC coating resistance to UV radiation, Fig. 3e shows the spectral stability of the coating after accelerated aging tests (Note S12). After 240 h of accelerated UV aging testing, the coating retains its original solar reflectance and mid-infrared emissivity, which indicates excellent resistance to potential UV degradation introduced by exposure to strong sunlight. This durability is largely attributed to the high UV reflectance of the HTCRC coating (i.e., approximately 95% at 340 nm), limiting UV penetration and suppressing structural degradation. Additional salt-spray corrosion tests were conducted to evaluate environmental durability under aggressive operating conditions. The HTCRC coating exhibited excellent salt-spray resistance, indicating its suitability for deployment on overhead transmission lines operating in saline and humid coastal environments (Note S13).

Finally, to ensure fire safety, especially under extreme environmental events, the HTCRC coating must exhibit effective flame-retardant behavior. Figure 3f shows that the coating does not ignite or deform when exposed to open flame, which is particularly important for preventing the propagation of fire along power lines in wildfire-prone regions (e.g., California, USA^45^). Together, these mechanical robustness, environmental durability, and fire-safety characteristics establish the foundation to evaluate the proposed HTCRC coating under realistic outdoor operating conditions.

### Experimental validation on cables in extreme heat conditions

**Fig. 4:**
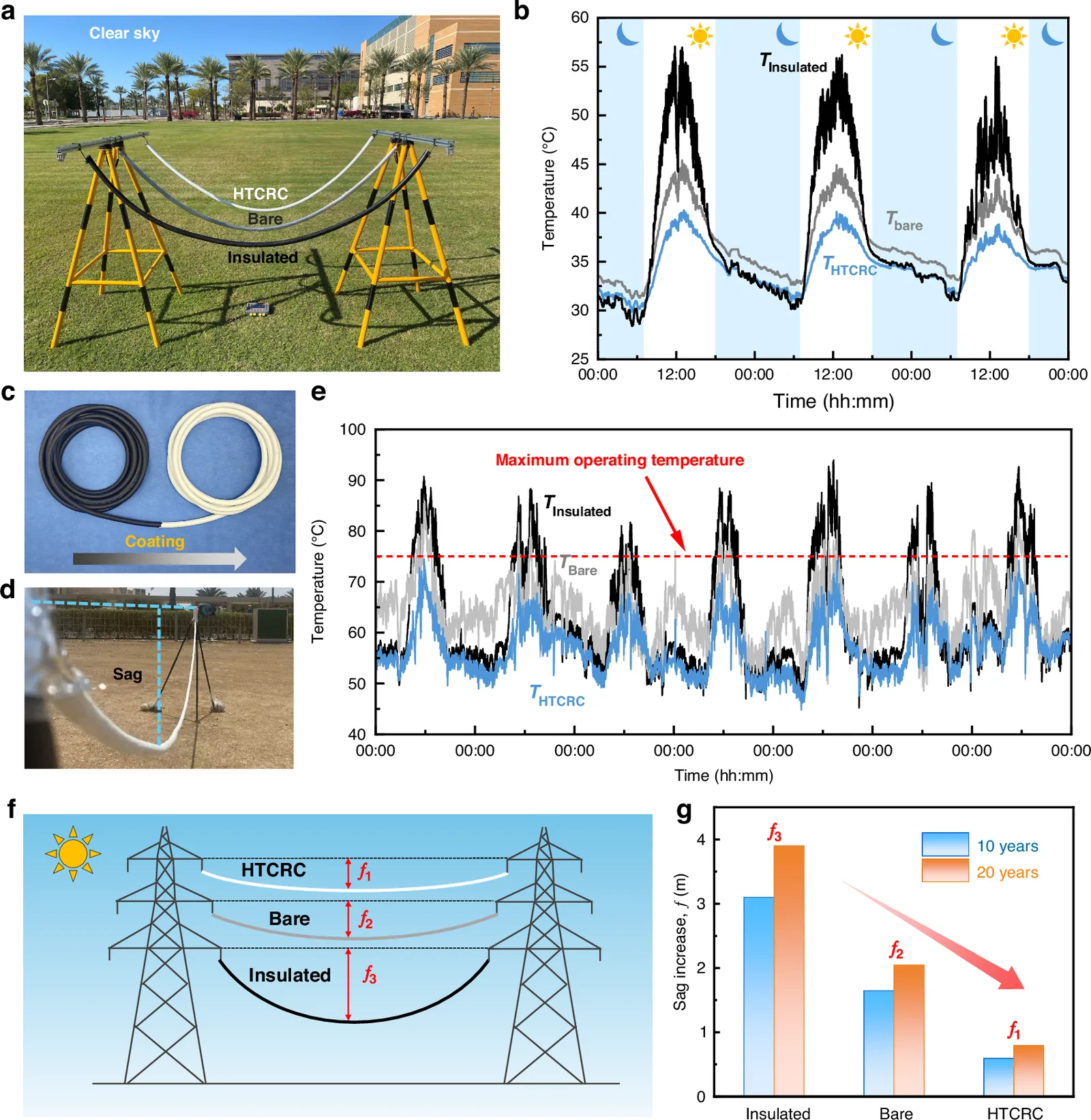
Outdoor demonstration of HTCRC-enabled thermal management for overhead cables. **a** Photograph of three suspended commercial ACSR cables with bare, insulated, and HTCRC-coated surfaces during outdoor field tests under no-current conditions. **b** Measured surface temperatures of the three cables under outdoor solar exposure. **c** Photograph of HTCRC coating applied to a simulated cable, demonstrating mechanical flexibility and surface conformity. **d** Initial sag of the HTCRC-coated cable. **e** Temperature profiles of the three cables over multiple day-night cycles. The HTCRC-coated cable consistently maintains the lowest temperature and remains below the maximum operating temperature of 75 °C, confirming its cooling effectiveness under realistic operating conditions. **f** Schematic of the catenary model to calculate cable sag. **g** Calculated sag increases of the three cables after 10 and 20 years

To validate the practical cooling performance of the proposed HTCRC coating in a real-world setting, we performed outdoor experiments on power cables under the extreme summer conditions of Saudi Arabia. As shown in Fig. 4a, we selected three representative cables: a commercially available insulated cable, a bare ACSR cable, and another commercial ACSR cable coated with the proposed HTCRC. The temperatures of the cables were continuously monitored during outdoor exposure under natural solar irradiation (refer to the technical details in Note S14). For safety regulation, the initial field measurements were performed under open circuit conditions. Over three days of continuous exposure (July 3 to July 5, 2025) in Thuwal, Saudi Arabia (Fig. 4b), the HTCRC-coated cable consistently remained the coolest among the three tested surfaces. In particular, the HTCRC-coated cable exhibited temperature reductions of ~5 °C and 15 °C compared with the bare and insulated cables, respectively, under peak midday solar irradiation of approximately 1100 W m^−2^. This performance reflects the ability of the proposed HTCRC coating to simultaneously suppress solar heat gain through high solar reflectance and dissipate heat through strong thermal infrared emission. Additional control experiments were conducted to distinguish the contribution of infrared radiative heat rejection from solar-reflection-induced cooling, confirming both the cooling effect associated with enhanced infrared emission and the sub-ambient cooling capability of the HTCRC coating under non-energized conditions (Notes S15 and S16).

To further examine cooling behavior under conditions involving internal heat generation, a set of simulated cables was designed and fabricated to replicate the geometry and Joule heating characteristics of actual overhead conductors while enabling safe outdoor operation at low applied currents. Figure 4c compares the simulated cables before and after the application of the proposed HTCRC coating. Here, internal heat was generated by Joule heating of the resistive wires embedded inside the simulated cables, thereby mimicking the heat generation of real-world overhead aluminum conductors (refer to the technical details in Note S17). Figure 4d shows the initial sag profile of the simulated HTCRC-coated cable, mimicking the cable configuration under self-weight conditions. Continuous temperature profiles over a representative seven-day measurement period (August 2 to August 8, 2025) were recorded, as shown in Fig. 4e (corresponding meteorological parameters in Note S18). Increased daytime wind speeds introduced fluctuations in the convective heat transfer. However, a clear and consistent performance trend emerged, i.e., both the insulated and bare simulated cables experienced rapid temperature increases, consistently exceeding the typical maximum safe operating temperature (~75 °C) for power conductors during peak solar irradiance (~1100 W/m^2^) and peak ambient temperatures of ~45 °C, reaching up to 80 °C and 90 °C, respectively. In stark contrast, the HTCRC-coated cable maintained a substantially lower temperature across the entire diurnal cycle, varying diurnally between 50 °C and 70 °C. Critically, even at midday, the HTCRC-coated cable remained ~5 °C below the safe operating temperature threshold, demonstrating robust cooling performance under intense solar exposure. The similar nighttime temperatures of the HTCRC-coated cable and the commercial insulated cable are physically expected because both surfaces exhibit similarly high mid-infrared emissivity (~0.95), enabling comparable infrared radiative heat rejection under nighttime conditions. In contrast, the bare aluminum cable possesses a much lower infrared emissivity (~0.1), which limits radiative heat rejection and leads to a higher nighttime operating temperature.

This reduction in operating temperature directly mitigates thermal expansion and, consequently, conductor sag, which is a critical factor for grid safety and reliability. From a mechanical perspective, conductor sag is quantitatively governed by the catenary profile, which describes the equilibrium shape of an overhead cable suspended under its own weight (Fig. 4f). When the surface heat transfer characteristics of the cable differ significantly, nonuniform operating temperatures can arise, leading to distinct thermal expansion levels and corresponding mechanical responses. These differences manifest as variations in the catenary geometry and, ultimately, different degrees of sag increase, as demonstrated by the three representative catenary profiles (*f*_1_, *f*_2_, and *f*_3_) corresponding to three cables in Fig. 4f.

To quantitatively capture this effect, the catenary equation, which is a standard analytical tool in transmission line engineering, was employed to model the long-term sag behavior (Note S19). The calculated results (Fig. 4g) predict the sag increase of the three cables after 10 and 20 years of service. As can be seen, the HTCRC-coated cable exhibits a markedly smaller sag increase than the other two cables. In addition, the projected increase in sag between years 10 and 20 is the lowest for the HTCRC-coated cable, indicating enhanced long-term dimensional stability. These findings demonstrate that the proposed HTCRC coating reduces operating temperatures and effectively suppresses sag, mitigating the risks of mechanical failure, short circuits, and power outages, thereby enhancing the long-term resilience and ampacity of power transmission infrastructure in hot climates. To bridge these benefits with practical transmission performance, we further assess how the reduced operating temperature translates into enhanced ampacity under realistic climatic conditions.

### Enhanced ampacity for increased global grid capacity

**Fig. 5:**
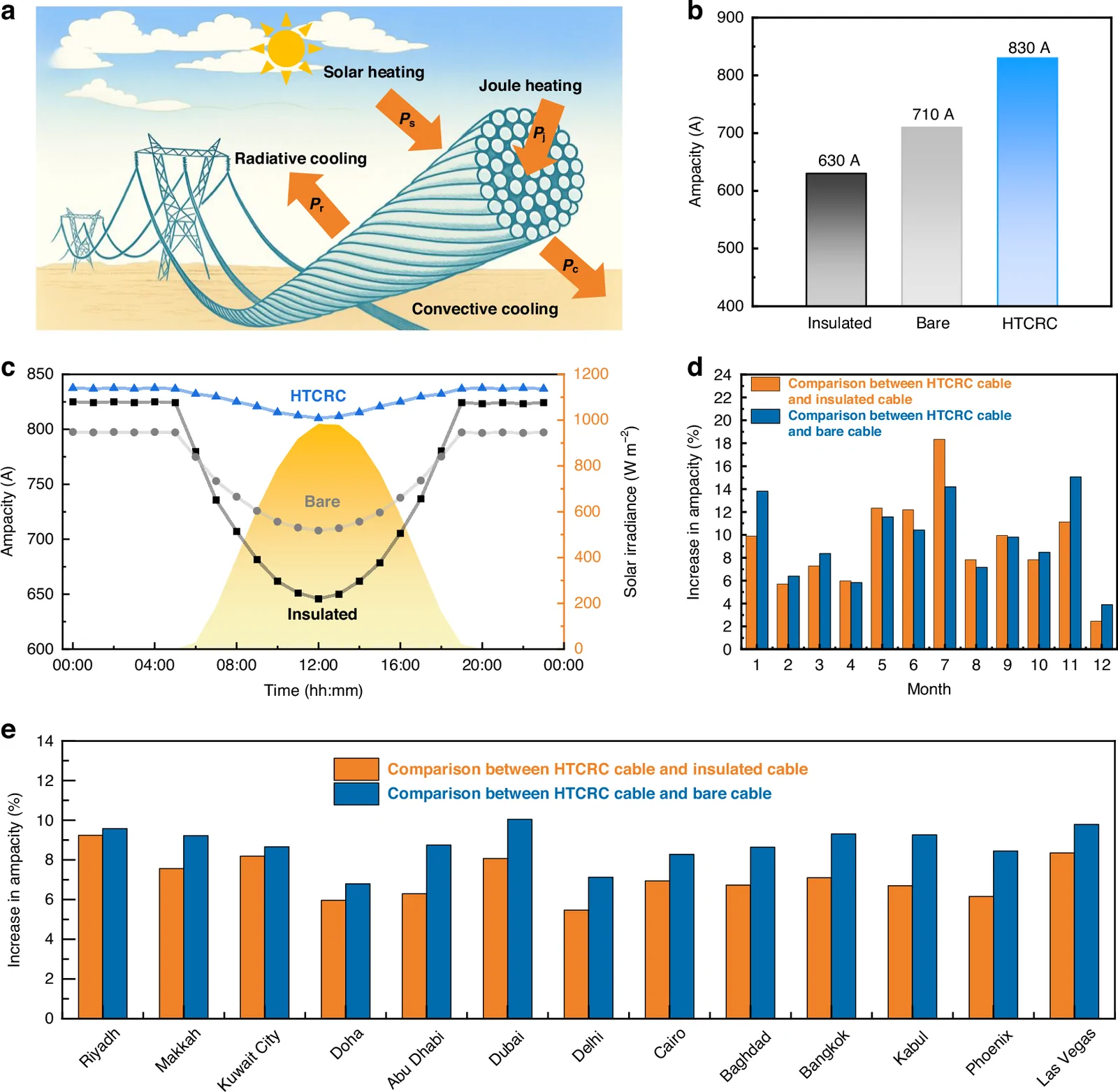
Ampacity enhancement enabled by proposed HTCRC coating. **a** Schematic of the cable heat balance model considering Joule heating, solar gain, convective cooling, and radiative cooling. **b** Simulated ampacity under constant ambient conditions, depicting HTCRC cables outperforming bare and insulated cables. **c** Diurnal variation in ampacity under dynamic solar irradiance, demonstrating thermal stability of the HTCRC cable. **d** Monthly ampacity improvement in Riyadh across a full year. **e** Simulated annual ampacity enhancement in 13 hot-climate cities worldwide, comparing HTCRC-coated cables with conventional bare and insulated conductors, demonstrating the broad applicability of the proposed coating technology

A key benefit of lower operating temperatures is enhanced ampacity, enabling higher current operation within thermal safety limits^13^. To quantify this critical performance metric, we employed the standard analytical heat balance model from CIGRE Technical Brochure 601^14,46^, which balances Joule heating *P*_j_ and solar heat gain *P*_s_ against convective *P*_c_ and radiative *P*_r_ cooling mechanisms (Fig. 5a, and refer to the technical details in Note S20). We applied this model to an ACSR 450 AlFe 8 conductor, which is widely used in high-voltage networks in hot climates^46^. Despite its stranded structure, the conductor can be reasonably approximated as having a uniform cross-sectional temperature because internal thermal resistance is negligible and previous simulations confirm that radial temperature differences remain below 4 K^47,48^. Based on this assumption, the ampacity can be reliably calculated to evaluate the effect of temperature reduction.

As shown in Fig. 5b, the HTCRC-coated cable demonstrates a striking improvement in current-carrying capacity under steady-state conditions. The calculated ampacity of the HTCRC-coated cable reaches 830 A, substantially exceeding that of the black insulated (630 A) and bare silver (710 A) cables. This advantage becomes even more pronounced under dynamic diurnal conditions that account for fluctuating solar irradiance (Fig. 5c, under conditions with peak ambient temperature and maximum solar irradiance of 40 °C and 1000 W m⁻^2^, respectively). Although the ampacity of the conventional cables considerably dropped during midday, the HTCRC-coated cable maintained remarkably stable performance. At solar noon, the HTCRC-coated cable achieved ampacity up to 165 and 102 A higher than the insulated and bare cables, respectively, which is directly attributable to its ability to minimize solar absorption while maximizing thermal emission.

This superior daytime performance is particularly critical for hot climates like in Saudi Arabia, where peak electricity demand, driven by air conditioning, coincides with maximum solar loading. Under such conditions, conventional conductors encounter a pronounced thermal bottleneck that directly compromises grid reliability. To evaluate this impact under realistic operating conditions, year-long performance was simulated for Riyadh, where summer temperatures frequently exceed 45 °C. The results reveal that the HTCRC-coated cable provides an average daytime ampacity increase of ~10% throughout the year, with a peak enhancement of 18.3% in July compared with the standard insulated cable (Fig. 5d). Extending this analysis globally to 13 representative hot cities reveals a potential annual daytime ampacity increase of 6%–10%, depending on local climatic conditions (Fig. 5e, and corresponding meteorological parameters in Note S21). Such enhancement considerably increases the transmission capacity of existing infrastructure without requiring costly or disruptive system upgrades. Thus, the proposed HTCRC technology offers a cost-effective (cost discussion in Note S22), passive, and scalable strategy to improve the efficiency and resilience of power grids, ensuring reliable energy delivery to satisfy the growing demands of a warming world.

## Discussion

This work establishes a quantitative, material-based pathway to relieve the fundamental thermal constraint imposed by excessive operating temperatures in urban power transmission infrastructure. By integrating high solar reflectance (0.92), strong mid-infrared emissivity (0.95), and elevated thermal conductivity (1.28 W m^−^¹ K^−^¹) within a single, retrofittable coating, the proposed HTCRC technology directly targets coupled heat transfer processes that govern the ampacity of overhead conductors. The results of practical outdoor experiments performed under extreme summer conditions in Saudi Arabia demonstrate that the HTCRC-coated cables maintained an operating temperature of 70 °C at an ambient temperature of 45 °C under full sun illumination of 1100 W/m^2^, which is well below the critical 75 °C safety threshold even under peak solar loading. When translated through CIGRE 601–based thermal modeling, this thermal margin corresponds to a projected ampacity increase of up to 18.3% during summer operation in Riyadh and a consistent 6%–10% enhancement across representative hot-climate cities worldwide.

Beyond these performance metrics, the broader significance lies in the system-level implications for cities facing simultaneous increases in electricity demand and ambient temperature, which considerably reduces the reliability of power systems under climate change and extreme heat conditions^2^. Urban power grids are increasingly constrained by a feedback loop, where rising ambient and operating temperatures degrades transmission capacity precisely when cooling-driven demand peaks^12^. By passively increasing ampacity, reducing resistive losses, and mitigating thermal sag and degradation without active control or infrastructure replacement, the proposed HTCRC coating offers a practical approach to decouple grid performance from extreme heat. This can defer costly grid expansion, reduce emissions associated with new construction, and enhance resilience in heat-stressed and rapidly urbanizing regions where overhead lines remain prevalent. More broadly, the findings of this study illustrate how quantitative gains achieved through surface-level materials engineering can unlock latent capacity in existing urban systems^49,50^. Such passive, scalable interventions will be increasingly vital for climate adaptation, enabling reliable electricity delivery as a foundation for sustainable urban development in a warming and electrifying world.

## Materials and methods

### Fabrication of HTCRC coating

The HTCRC coating was fabricated by dispersing h-BN particles (Maclin, China) into a PDMS precursor (Sylgard 184, Dow Corning, USA). Unless otherwise specified, the HTCRC coatings used in the outdoor cooling experiments, simulated cable experiments, and durability tests contained 40 vol% h-BN. To ensure adequate dispersion and processability, ethyl acetate was introduced as a diluent. The mass ratio of PDMS component A, component B, and ethyl acetate was maintained at 2:10:1.5. The resulting mixture was subjected to vacuum degassing using a centrifugal mixer operating at 2000 rpm for 15 min to eliminate entrapped air bubbles. Once homogeneously mixed and degassed, the composite precursor was applied to the target surface as a uniform coating. The applied coating was then thermally cured in an oven at 60 °C for 2 h to obtain the final HTCRC coating. A coating thickness of ~2.8 mm was adopted in the experimental demonstrations unless otherwise specified.

### Characterizations

The morphology of the h-BN particles and the cross-sectional structure of the HTCRC coating were characterized using a field emission scanning electron microscope (Thermo Fisher Scientific Teneo, USA) operating at an acceleration voltage of 5 kV. The solar spectral reflectance ${R}_{{{\rm{solar}}}}$ of the coatings was measured using a spectrophotometer (LAMBDA 950, PerkinElmer, USA) equipped with an integrating sphere, covering a spectral range from 400–2500 nm. The mid-infrared reflectance ${R}_{{{\rm{IR}}}}$ and transmittance ${T}_{{{\rm{IR}}}}$ were measured using a Fourier transform infrared spectrometer (Bruker Vertex 80) coupled with a gold-plated integrating sphere over the spectral range of 3–14 µm. To minimize interference from atmospheric moisture and carbon dioxide during the infrared measurements, the spectrometer chamber was purged with dry nitrogen before testing. The total mid-infrared emissivity ${E}_{{{\rm{IR}}}}$ of the coating was calculated based on Kirchhoff’s law and the principle of energy conservation using the relation ${E}_{{{\rm{IR}}}}={A}_{{{\rm{IR}}}}=1-{R}_{{{\rm{IR}}}}-{T}_{{{\rm{IR}}}}$, where ${A}_{{{\rm{IR}}}}$ denotes the mid-infrared absorptance of HTCRC coating. The thermal conductivity of the HTCRC coating was evaluated using the Hot Disk thermal constants analyzer (TPS 2500 S) following the transient plane source method. h-BN particles with different sizes were obtained by processing them using a ball mill, and the particle size distribution was measured using a laser particle size analyzer (Jnwinner-802).

### Durability tests

The tensile properties of the coating were evaluated using a universal testing machine, with the upper fixture moving upward at a constant rate of 5 mm/min. The surface hydrophobicity was characterized using a contact angle goniometer (DSA100E, KRÜSS GmbH, Germany) by measuring the static water contact angle on the coating surface. To examine UV durability, an accelerated aging test was conducted according to ASTM G154-23 using a UVA-340 weathering chamber. The total exposure duration was 240 h. Each cycle consisted of 8 h of UVA-340 irradiation (0.89 ± 0.02 W m⁻² nm⁻¹ at 340 nm, black panel temperature: 60 ± 3°C), followed by 4 h of condensation at a black panel temperature of 50 ± 3 °C.

### Outdoor cooling demonstration

To monitor the thermal performance of the samples under real-world conditions, outdoor experiments were conducted at the experimental field site at King Abdullah University of Science and Technology. The temperatures of the samples were measured using calibrated K-type thermocouples with a resolution of ±0.1 °C. Each thermocouple was connected to a multichannel data acquisition system for continuous temperature logging. The environmental conditions, such as global horizontal irradiance, ambient temperature, and wind speed, were recorded simultaneously using a NEO Meteo-40 weather station. For the simulated cable experiments, the nickel-chromium alloy resistance wires inside each cable were powered to provide a total heating load of 30 W per cable, replicating the Joule heating experienced during actual cable operation.

## Supplementary information

SUPPLEMENTAL MATERIAL

## Data Availability

Data supporting the findings of this study are available within the article and its Supplementary Information. Additional data are available from the corresponding author upon reasonable request.
