# Supplementary material for: Photonic thermal management of overhead cables via high-thermal-conductivity radiative cooling
Source: Light Sci Appl. 2026 Oct 8;15:385. doi: 10.1038/s41377-026-02485-1 (PMC13645785; doi:10.1038/s41377-026-02485-1)
Supplement: Supplementary file 1 [file 41377_2026_2485_MOESM1_ESM.docx]

**SUPPLEMENTARY INFORMATION FOR**

**Photonic thermal management of overhead cables via high-thermal-conductivity radiative cooling**

Kai Xu^1,2^, Peng-Fei Cao^3^, Jehad K. El-Demellawi^2^, Hussam Qasem^4^, Qiaoqiang Gan^1^^,2^ *

^1^*Division of Physical Science and Engineering, King Abdullah University of Science and*

*Technology (KAUST), Thuwal 23955-6900, Saudi Arabia.*

^2^*Center for Renewable Energy and Storage Technologies (CREST), King Abdullah University of Science and Technology (KAUST), Thuwal 23955-6900, Saudi Arabia.*

^3^*State Key Laboratory of Organic-Inorganic Composites, College of Materials Science and Engineering, Beijing University of Chemical Technology, Beijing 100029, China**.*

^4^*Future Energy Technology Institute, King Abdulaziz City for Science and Technology, Riyadh 11442, Saudi Arabia.*

*Correspondence to: Q.G. (E-mail: [qiaoqiang.gan@kaust.edu.sa](mailto:qiaoqiang.gan@kaust.edu.sa))

**Figure S1.** Individual contributions of conductive, convective, and radiative thermal resistances as a function of coating thickness.

**Figure S2.** Dependence of the critical thickness on wind speed for different cable radii, compared with the IEC 60502-2 recommended insulation thickness.

**Note S1. Thermal resistance model and critical insulation radius**

To quantitatively evaluate this effect, we constructed a thermal resistance network model, which accounts for the primary heat transfer pathways in an overhead cable: thermal conduction through the HTCRC layer, with subsequent heat dissipation via convection and radiation at the surface. The total thermal resistance $R_{\text{tot}}$ is expressed as:

$$R_{\text{tot}}=R_{\text{cond}}+\frac{1}{\frac{1}{R_{\text{conv}}}+\frac{1}{R_{\text{rad}}}}$$

where$R_{\text{cond}}$,$R_{\text{conv}}$, and $R_{\text{rad}}$represent the conductive, convective, and radiative thermal resistances, respectively.

where $r_{1}$ is the radius of the bare conductor, and $r_{2}$ is the radius of the cable after applying the HTCRC coating. *L* is the cable length, *k* is the thermal conductivity of the HTCRC coating, *h* is the convective heat transfer coefficient, *h*_r_ is the radiative heat transfer coefficient, *A* is the surface area of the cable, *ε* is the emissivity, *σ* is the Stefan–Boltzmann constant, *T*_s_ is the cable surface temperature, and *T*_sur_ is the ambient temperature. The radius $r_{2}$ at which $R_{\text{tot}}$ reaches its minimum is defined as the critical insulation radius$r_{\text{cr}}$.

**Note S2. Heat-flux decomposition analysis of cable heat dissipation**

To quantitatively evaluate the relative contributions of convection and radiation to cable heat dissipation, a heat-transfer analysis was performed under representative operating conditions. The coating thickness was set to 2.8 mm. To isolate the effect of radiative heat dissipation and eliminate the influence of solar irradiation, the analysis was conducted under nighttime conditions. The ambient wind speed was set to 1 m s⁻¹, corresponding to a representative low-wind nighttime condition in Jeddah, Saudi Arabia, one of the target application environments considered in this work. The Joule heating power of the conductor was fixed at 100 W m⁻¹. The calculated convective heat flux, radiative heat flux, and operating temperatures of the three cable configurations are summarized in **Table. S1**. The results show that both the HTCRC-coated cable and the insulated cable exhibit radiative heat dissipation of approximately 27 W m⁻¹, accounting for nearly 27% of the total heat rejection. By comparison, the bare conductor exhibits a radiative heat loss of only 4.0 W m⁻¹, with heat dissipation occurring predominantly through convection. Consequently, the operating temperature of the bare conductor reaches approximately 65.1°C, whereas the HTCRC-coated cable operates at a substantially lower temperature of approximately 59.1°C. The similarity between the HTCRC-coated cable and the insulated cable is physically reasonable because both surfaces possess high infrared emissivity (~0.95), enabling efficient radiative heat dissipation and resulting in comparable thermal performance under nighttime conditions. In contrast, the low-emissivity bare conductor is unable to effectively utilize radiation as a heat rejection pathway, requiring a much larger temperature difference relative to the environment to dissipate the same Joule heat load.

**Table S1.** Convective and radiative heat losses of bare, insulated, and HTCRC-coated cables

| Cable | Convective heat loss  (W m⁻¹) | Radiative heat loss  (W m⁻¹) | Temperature  (°C) |
| --- | --- | --- | --- |
| HTCRC | 73.7 | 26.3 | 58.7 |
| Insulated | 72.3 | 27.7 | 59.1 |
| Bare Al | 96.0 | 4.0 | 65.1 |

Therefore, although convection remains the dominant heat dissipation mechanism under most practical operating conditions, the relatively larger radiative thermal resistance does not imply that radiation is negligible. On the contrary, the present analysis demonstrates that enhanced infrared emissivity can provide a substantial additional heat dissipation channel, particularly under low-wind conditions where convective cooling becomes less effective. Together with suppressed solar absorption and improved conductive heat spreading, this enhanced radiative heat dissipation contributes significantly to the overall cooling performance of the HTCRC coating.

**Note S3. Lorenz**–**Mie scattering model**

The solar reflectance of the HTCRC coating originates from the interaction between incident sunlight and embedded h-BN particles. To quantify this interaction, the optical response of individual particles was first analyzed and subsequently linked to the macroscopic radiative behavior of the composite coating. Because the characteristic dimensions of the h-BN particles are comparable to the wavelengths within the solar spectrum (250–2500 nm), the scattering process falls within the Mie-scattering regime. Therefore, Lorenz–Mie theory was adopted to evaluate the scattering and absorption characteristics of the particles [1]. Within this framework, particle radiative behavior is governed by the size parameter and the complex refractive-index ratio. Based on these parameters, the extinction efficiency, scattering efficiency, absorption efficiency, and phase function can be determined. The scattering properties are related to the size parameter and the dimensionless complex refractive index parameter [2]. The expression is given by:

$$m=(n+i\kappa)/n_{0}$$

where *n* and$\kappa$ are the refractive index and extinction coefficient of the particle, respectively. The attenuation efficiency $Q_{\text{ext}}$, scattering efficiency $Q_{\text{sca}}$, absorption efficiency $Q_{\text{abs}}$, and scattering phase function $\Phi(\Theta)$ are used to describe the radiative properties of particles. Among them, the first three parameters represent extinction, scattering, and absorption cross-sections normalized by the particle's geometric cross-sectional area. The following relationship holds:

$$Q_{\text{ext}}=Q_{\text{sca}}+Q_{\text{abs}}$$

Under the Lorentz–Mie scattering model, $Q_{\text{sca}}$ and $Q_{\text{ext}}$ are expressed as:

$$Q_{\text{sca}}=\frac{2}{\chi^{2}}\sum_{k=1}^{\infty} (2k+1)(\left|a_{k}\right|^{2}+\left|b_{k}\right|^{2})$$

$$Q_{\text{ext}}=\frac{2}{\chi^{2}}\sum_{k=1}^{\infty} (2k+1)\mathrm{Re}(a_{k}+b_{k})$$

where Re denotes the real part, and *χ* is size parameter. The Mie coefficients $a_{k}$ and $b_{k}$ represent the electric and magnetic multipole amplitudes of order *k*, respectively:

$$a_{k}=\frac{m\psi_{k}(m\chi)\psi'_{k}(\chi)-\psi'_{k}(m\chi)\psi_{k}(\chi)}{m\psi_{k}(m\chi)\xi'_{k}(\chi)-\psi'_{k}(m\chi)\xi_{k}(\chi)}$$

$$b_{k}=\frac{m\psi'_{k}(m\chi)\psi_{k}(\chi)-\psi_{k}(m\chi)\psi'_{k}(\chi)}{m\psi'_{k}(m\chi)\xi_{k}(\chi)-\psi_{k}(m\chi)\xi'_{k}(\chi)}$$

$$\xi_{k}=\psi_{k}-i\xi_{k}$$

$\psi_{k}(\chi)$ and $\xi_{k}(\chi)$ are Riccati–Bessel functions of order *k*, which are related to the spherical Bessel functions of the first and second kinds, $j_{k}(\chi)$and$y_{k}(\chi)$, respectively:

$$\psi_{k}(\chi)=\chi j_{k}(\chi)$$

$$\xi_{k}(\chi)=\chi\left[j_{k}(\chi)-iy_{k}(\chi)\right]$$

The summation index *k* should ideally run from 1 to ∞, but Bohren and Huffman proposed a practical upper truncation limit $k_{\max}$. By truncating the infinite sum at $k_{\max}$, the influence of later terms can be neglected, which helps reduce computational time. The formula for $k_{\max}$is given as [2]:

$$k_{\max}=\chi+4.3\chi^{1/3}+1$$

Here, $k_{\max}$ is an integer, and the result is rounded to the nearest multiple of 5 for symmetrical computation. $k=2$represents electric and magnetic quadrupoles, corresponding to Rayleigh scattering; $k=4$ represents electric and magnetic hexadecapoles, falling under Mie scattering. The scattering phase function $\Phi(\Theta)$describes the angular distribution of scattered radiation, representing the ratio of scattered intensity in direction $\Theta$ to the incident intensity. It characterizes the angular scattering properties of the particle under long-wavelength illumination. In $\Phi(\Theta)$, $\Theta$ denotes the scattering angle between the incident and scattered light. Forward scattering corresponds to $\Theta<90^{\circ}$, and backward scattering corresponds to $\Theta>90^{\circ}$. The expression of the phase function is:

$$\Phi(\Theta)=2\frac{\left|S_{1}\right|^{2}+\left|S_{2}\right|^{2}}{\chi^{2}Q_{\text{sca}}}$$

Here, $S_{1}$ and $S_{2}$ are the complex scattering amplitudes for perpendicular and parallel polarized components, respectively. They are expressed as:

$$S_{1}(\cos\Theta,\text{ }m,\text{ }\chi)=\sum_{k=1}^{\infty} \frac{(2k+1)}{k(k+1)}(a_{k}\pi_{k}+b_{k}\tau_{k})$$

$$S_{2}(\cos\Theta,\text{ }m,\text{ }\chi)=\sum_{k=1}^{\infty} \frac{(2k+1)}{k(k+1)}(b_{k}\pi_{k}+a_{k}\tau_{k})$$

where $\cos\Theta$ is the cosine of the scattering angle. $\pi_{k}$ and $\tau_{k}$ are angular scattering modes of the spherical harmonics, and their relations to the Legendre polynomial $P_{k}$ are given as:

$$\pi_{k}(\cos\Theta)=\frac{P_{k}^{1}(\cos\Theta)}{\sin\Theta}=\frac{\text{d}P_{k}(\cos\Theta)}{\text{d}\cos\Theta}$$

$$\tau_{k}(\cos\Theta)=\frac{\text{d}P_{k}^{1}(\cos\Theta)}{\text{d}\Theta}=\cos\Theta\pi_{k}(\cos\Theta)-\sin^{2}\Theta\frac{\text{d}\pi_{k}(\cos\Theta)}{\text{d}\cos\Theta}$$

The expression for $P_{k}$ is:

$$P_{k}^{1}(\cos\Theta)=-\sin\Theta\frac{\text{d}P_{k}(\cos\Theta)}{\text{d}\cos\Theta}$$

The asymmetry of the scattering direction can be represented by the asymmetry factor *g*, which physically corresponds to the average value of the cosine of the scattering angle. The expression is given as [2]

$$g=\overline{\cos\Theta}=\frac{1}{4\pi}\int_{4\pi} \Phi(\Theta)\cos\Theta\mathrm{d}\Theta=\frac{1}{2}\int_{0}^{\pi} \Phi(\Theta)\cos\Theta\sin\Theta\mathrm{d}\Theta$$

The value of *g* ranges between [-1, 1]. When the particles scatter light isotropically and symmetrically about the direction perpendicular to the incident light, $g=0$. When$\Theta<\pi/2$, forward scattering dominates and $g>0$; conversely, when backward scattering dominates, $g<0$.

The HTCRC coating can be regarded as a heterogeneous medium consisting of h-BN particles dispersed within a PDMS matrix. Under the assumption that particle–particle interactions are negligible and each particle scatters independently, the bulk optical properties of the coating can be obtained by summing the contributions from particles of different sizes [3]. Using the experimentally measured particle-size distribution, the ensemble scattering coefficient $\sigma_{\text{p}}$, absorption coefficient $\alpha_{\text{p}}$, hemispherical reflectance fraction $F_{\text{p}}$, and asymmetry factor $g_{\text{p}}$ are calculated from the corresponding single-particle quantities as follows [1, 4]:

$$\sigma_{\text{p}}=\sum_{i=1}^{n} \frac{\pi}{4}D_{i}^{2}N_{i}Q_{\text{sca, }i}=\sum_{i=1}^{n} \frac{\pi}{4}D_{i}^{2}\frac{6f_{v,\text{ }i}}{\pi D_{i}^{3}}Q_{\text{sca, }i}=1.5\sum_{i=1}^{n} Q_{\text{sca, }i}\frac{f_{v,\text{ }i}}{D_{i}}$$

$$\alpha_{\text{p}}=\sum_{i=1}^{n} \frac{\pi}{4}D_{i}^{2}N_{i}Q_{\text{abs, }i}=\sum_{i=1}^{n} \frac{\pi}{4}D_{i}^{2}\frac{6f_{v,\text{ }i}}{\pi D_{i}^{3}}Q_{\text{abs, }i}=1.5\sum_{i=1}^{n} Q_{\text{abs, }i}\frac{f_{v,\text{ }i}}{D_{i}}$$

$$F_{\text{p}}=\frac{\int_{0}^{\pi/2} \Phi(\cos\theta)_{\text{p}}\sin\theta\mathrm{d}\theta}{\int_{0}^{\pi} \Phi(\cos\theta)_{\text{p}}\sin\theta\mathrm{d}\theta}$$

$$g_{\text{p}}=\frac{1.5}{\sigma_{\text{p}}}\sum_{i=1}^{n} \frac{f_{v,\text{ }i}g_{\mathrm{s},\text{ }i}Q_{\text{sca},\text{ }i}}{D_{i}}$$

The units of $\sigma_{\text{p}}$ and $\alpha_{\text{p}}$ are both $\text{m}^{-1}$. The subscript *i* denotes particles of different diameters within the particle ensemble. $N_{i}$ and $f_{v,\text{ }i}$ represent the number density and volume fraction of particles with diameter $D_{i}$, respectively. The attenuation coefficient can be obtained using the relation:. When particles of various compositions and sizes are uniformly dispersed in the same matrix, they are referred to as multicomponent particles. Assuming that these multicomponent particles are uniformly distributed within the matrix and that neighboring particles scatter independently, the scattering and absorption coefficients of the particle ensemble follow a linear combination of their respective components [5]. In this case, the scattering coefficient $\sigma$ and total absorption coefficient $\alpha$ of the particle-embedded composite can be calculated using the following formula:

$$\sigma=\sigma_{\text{p}}=\sum_{j=1}^{N} c_{\text{p},j}\sigma_{\text{p},j}$$

$$\alpha=\alpha_{\text{p}}+\alpha_{\text{b}}=\sum_{j=1}^{N} c_{\text{p},j}\alpha_{\text{p},j}+(1-c_{\text{p}})\alpha_{\text{b}}$$

In these equations, $c_{\text{p},j}$ , $\sigma_{\text{p},j}$, and $\alpha_{\text{p},j}$ denote the volume fraction, scattering coefficient, and absorption coefficient of the *j* type of particle, respectively. *N* is the number of particle types, and $\alpha_{\text{b}}$ is the absorption coefficient of the matrix material.

**Note S4. Refractive-index parameters used in optical calculations**

The optical calculations were performed using the wavelength-dependent refractive index (*n*) and extinction coefficient (*k*) of h-BN. The refractive index of the PDMS matrix, *n*_0_, was set to 1.42. The h-BN optical constants used in the Lorenz–Mie scattering calculations and Monte Carlo optical simulations are shown in **Fig. S3**.

**Figure S3.** Wavelength-dependent refractive index (*n*) and extinction coefficient (*k*) of h-BN used in the optical scattering simulations.

**Note S5. Monte Carlo model**

A stochastic photon-tracing approach based on the Monte Carlo method was employed to predict the optical reflectance of the HTCRC coating. In this approach, a large number of photons are launched into the coating and individually tracked while undergoing absorption, scattering, reflection, and transmission events. The collective behavior of these photons is then used to determine the macroscopic optical response of the material. For each photon, the propagation distance is randomly sampled according to the extinction coefficient of the medium. Upon encountering an interface, reflection and transmission probabilities are evaluated using the corresponding optical boundary conditions. Photon energy is continuously attenuated through absorption within the coating, while scattering events modify the propagation direction according to the prescribed phase function. Photons are followed until they either leave the computational domain or are terminated using the Russian-roulette criterion. The overall reflectance is obtained from the fraction of photon energy exiting through the incident side of the coating.

The numerical implementation follows the radiative-transfer procedure is described below. First, the total number of photons *N*, initial energy, launch position, and propagation direction are specified. The photon number must be sufficiently large to ensure computational accuracy. A Cartesian coordinate system is adopted with the coating-thickness direction defined as the z-axis. Assuming the initial emission energy of a photon is $W=1$, and the emission position is $(x\text{, }y\text{, }z)$, the emission direction is represented by a unit vector $\vec{r}$. The directional cosines $(\mu_{x},\text{ }\mu_{y},\text{ }\mu_{z})$ of the photon can then be expressed as:

$$\begin{matrix} \mu_{x}=\vec{r}\cdot x \\ \mu_{y}=\vec{r}\cdot y \\ \mu_{z}=\vec{r}\cdot y \end{matrix}$$

where *x*, *y*, and *z* are unit vectors along each coordinate axis. The emission position of the photon is generally defined in Cartesian coordinates. The coordinate origin is set as $(0,\text{ }0,\text{ }0)$, and the directional cosines are defined as $(0,\text{ }0,\text{ 1})$ [6]. When a photon is reflected at the interface from a medium, part of its energy is lost due to reflection, resulting in an updated energy:

$$W=1-r_{\text{c}}$$

where $r_{\text{c}}$ is the interface reflectance. Afterward, a random number is generated to sample the photon's path length, assuming the photon propagates through the medium with exponentially distributed free paths. Assuming the photon travels in a medium with no boundary loss, the extinction coefficient $\mu_{\text{t}}$ (representing the probability of photon-medium interaction per unit path length) is defined as:

$$\mu_{\text{t}}=\frac{-\text{d}P\left\{s\geq s'\right\}}{P\left\{s\geq s'\right\}\text{d}s'}$$

where *P* is the probability of occurrence. Assuming the photon travels a step length $s_{1}$ , integrating over $s'$ in $(0,\text{ }s_{1})$ , we obtain:

$$P\left\{s\geq s_{1}\right\}=e^{-\mu_{\text{t}}s_{1}}$$

Therefore:

$$P\left\{s<s_{1}\right\}=1-e^{-\mu_{\text{t}}s_{1}}$$

The corresponding probability density function within the path length *s* is:

$$p(\mathrm{s}_{1})=\frac{\text{d}P\left\{s<s_{1}\right\}}{\text{d}s_{1}}=\mu_{\text{t}}e^{-\mu_{\text{t}}s_{1}}$$

Here, we assume the photon's step length *s*, where interaction with the medium occurs, is a random variable. Its probability density function is$p(s)$, defined over the interval$(a,\text{ }b)$. Using a computer to generate a random number $\xi$ uniformly distributed in $(0,\text{ 1})$, the inverse transform sampling method can be used to determine *s* as follows:

$$\int_{a}^{\chi} p(\chi)\mathrm{d}\chi=\xi$$

Combining Eq. (34) and Eq. (35), we obtain:

$$s=-\text{In}\xi\text{/}\mu_{t}$$

After obtaining the photon step length *s*, the particle position is updated as:

$$\begin{matrix} x=x+\mu_{x}s \\ y=y+\mu_{y}s \\ z=z+\mu_{z}s \end{matrix}$$

During the photon’s propagation, it may encounter the upper or lower boundary of the medium, where reflection or transmission occurs. The distance $d_{\text{b}}$ from the current photon position to the boundary in the direction of photon propagation is:

$$d_{\text{b}}\left\{\begin{matrix} \frac{z_{0}-z}{\mu_{z}}\text{ }if\text{ }\mu_{z}<0 \\ \inf\text{ }if\text{ }\mu_{z}=0\text{ } \\ \frac{z_{1}-z}{\mu_{z}}\text{ }if\text{ }\mu_{z}>0 \end{matrix}\right.$$

Here, $z_{0}$ and $z_{1}$ are the positions of the lower and upper boundaries of the medium layer. If $s<d_{\text{b}}\mu_{\text{t}}$, the photon will not encounter the interface. If $s\geq d_{\text{b}}\mu_{\text{t}}$, the photon will collide with the interface, and its position needs to be updated. The angle of incidence $\alpha_{1}$ at the boundary is calculated from the photon’s directional cosine:

$$\alpha_{1}=\cos^{-1}(\left|\mu_{z}\right|)$$

Photon transmission and reflection at the interface are described using Fresnel's equation [7]:

$$R=\frac{1}{2}\left(\left|\frac{n_{1}\cos\alpha_{2}-n_{2}\cos\alpha_{1}}{n_{1}\cos\alpha_{2}+n_{1}\cos\alpha_{2}}\right|^{2}+\left|\frac{n_{1}\cos\alpha_{1}-n_{2}\cos\alpha_{2}}{n_{1}\cos\alpha_{1}+n_{2}\cos\alpha_{2}}\right|^{2}\right)$$

where $n_{1}$ and $n_{2}$ are the refractive indices of the medium and air, respectively. $\alpha_{1}$ and $\alpha_{2}$ are the angles of incidence and refraction, related by:

$$n_{1}\sin\alpha_{1}=n_{2}\sin\alpha_{2}$$

A random number $\xi$ is uniformly sampled in the interval $(0,\text{ }1)$. When $\xi>R$, transmission occurs and the photon's trajectory is terminated. If the reflection occurs at the upper boundary, it is recorded as upward reflection; if at the lower boundary, it is recorded as downward transmission:

$$R_{\text{u}}=R_{\text{u}}+W\text{ }if\text{ }z=z_{0}$$

$$T_{\text{d}}=T_{\text{d}}+W\text{ }if\text{ }z=z_{1}$$

If $\xi<R$ , internal reflection occurs, and the directional cosine of the photon is updated as:

$$(\mu_{x},\text{ }\mu_{y},\text{ }\mu_{z})=(\mu_{x},\text{ }\mu_{y},\text{ }-\mu_{z})$$

Sampling is performed based on the absorption and scattering coefficients. When a photon is absorbed, its propagation continues in the original direction over a new distance. When a photon is scattered, the scattering angle is sampled to assign a new propagation direction and distance. When a photon travels a step of length *s*, the energy loss due to absorption by the medium is:

$$\Delta W=W\mu_{a}/\mu_{t}$$

The energy carried by the photon is then updated as:

$$W=W-\Delta W$$

The lost energy is assigned to the grid cell *A* corresponding to the particle path:

$$A=A+\Delta W$$

After traveling a distance *s*, the photon undergoes scattering, and its direction changes. Assume the scattering polar angle is $\theta_{\text{p}}$, taking values in $[0,\text{ }\pi)$, and the azimuthal angle is $\psi_{\text{p}}$, taking values in $[0,\text{ 2}\pi)$. The probability density distribution of the directional cosine $\cos\theta_{\text{p}}$ follows the Henyey–Greenstein phase function [8]:

$$p(\cos\theta)=\frac{1-g^{2}}{2(1+g^{2}-2g\cos\theta_{\text{p}})}$$

Here, $\cos\theta_{\text{p}}$ can be expressed using a random variable $\xi$ :

$$\cos\theta_{\text{p}}=\left\{\begin{matrix} \frac{1}{2g}\left[1+g^{2}-\left(\frac{1-g^{2}}{1-g+2g\xi}\right)^{2}\right]\text{ if g > 0} \\ 2\xi-1\text{ if g = 0} \end{matrix}\right.$$

The azimuthal angle $\psi_{p}$ can be expressed using a random number $\xi$ as:

$$\psi_{p}=2\pi\xi$$

The updated directional cosines of the photon after scattering are given by:

$$\mu'_{x}=\frac{\sin\theta_{\text{p}}}{\sqrt{1-\mu_{z}^{2}}}(\mu_{x}\mu_{z}\cos\psi_{p}-\mu_{y}\sin\psi_{p})+\mu_{x}\cos\theta_{p}$$

$$\mu'_{y}=\frac{\sin\theta_{\text{p}}}{\sqrt{1-\mu_{z}^{2}}}(\mu_{y}\mu_{z}\cos\psi_{p}-\mu_{x}\sin\psi_{p})+\mu_{y}\cos\theta_{p}$$

$$\mu'_{z}=-\sin\theta_{\text{p}}\cos\psi_{\text{p}}\sqrt{1-\mu_{z}^{2}}+\mu_{z}\cos\theta$$

It is worth noting that when the photon direction is nearly parallel to the *z*-axis (), the directional cosines are updated as:

$$\mu'_{x}=\sin\theta_{\text{p}}\cos\psi_{\text{p}}$$

$$\mu'_{\text{y}}=\sin\theta_{\text{p}}\sin\psi_{\text{p}}$$

$$\mu'_{z}=\text{SIGN}(\mu_{z})\cos\theta_{\text{p}}$$

In the expression, $\text{SIGN}(\mu_{z})$ takes the value 1 when $\mu_{z}>0$ , and –1 when $\mu_{z}<0$. Finally, the photon's direction is updated as $\mu_{x}=\mu'_{x}$, $\mu_{y}=\mu'_{y}$, and $\mu_{z}=\mu'_{z}$.After undergoing the various processes described above, the photon’s energy may decrease to a negligible level, at which point its motion should terminate. However, to conserve total energy even for photons with insufficient energy for interactions, the **Russian roulette** method is adopted [9]. This method probabilistically terminates photon paths without violating energy conservation. Given a threshold energy $W_{\text{th}}$ and roulette parameter $m_{1}$, when the photon’s energy weight drops below $W_{\text{th}}$, and a randomly sampled number , the photon survives and its weight is updated to $m_{1}W$; otherwise, it is terminated with weight reduced to zero:

$$W=\left\{\begin{matrix} m_{1}W\text{ }if\text{ }\xi\leq1/m_{1} \\ 0\text{ }if\text{ }\xi>1/m_{1} \end{matrix}\right.$$

After all emitted photons have stopped propagating, the HTCRC coating’s reflectance is calculated as:

$$R=R_{\text{u}}/NW$$

**Note S6. Reliability assessment of the optical scattering model**

The Lorenz–Mie analysis employed in this work was intended to provide physical guidance for identifying the particle-size range that maximizes solar scattering efficiency. It should be noted that the model is strictly derived for isolated spherical particles, whereas the h-BN fillers used in this work exhibit platelet-like morphology and may partially aggregate within the composite coating. Therefore, the calculated results should be interpreted as qualitative design guidance rather than exact prediction of the optical response. Nevertheless, previous studies have shown that Mie-based calculations can still provide useful guidance for identifying dominant scattering trends and optimal particle-size ranges in systems containing randomly oriented irregular particles [10]. To experimentally validate the model predictions, HTCRC coatings containing h-BN fillers with different characteristic particle sizes were prepared and characterized (**Fig. S4**). The experimental results show that coatings containing h-BN fillers with characteristic particle sizes around 400 nm exhibit the highest solar reflectance, consistent with the trends predicted by the Lorenz–Mie analysis and Monte Carlo simulations. This agreement confirms that the model captures the dominant scattering behavior and provides useful guidance for the optical design of HTCRC coatings.

**Figure S4.** Comparison between calculated and experimentally measured solar reflectance of HTCRC coatings containing h-BN fillers with different average particle sizes.

**Note S7. Comparison with representative radiative cooling materials**

To clarify the application-specific performance of the HTCRC coating, its solar reflectance, mid-infrared emissivity, and thermal conductivity were compared with representative radiative cooling materials reported in previous studies. As summarized in **Table S2**, conventional radiative cooling coatings can achieve high solar reflectance and strong mid-infrared emissivity, but they typically exhibit relatively low thermal conductivity because they are mainly designed for planar passive cooling applications, such as building envelopes. In contrast, the HTCRC coating is designed for internally heated overhead conductors, where efficient heat transport through the coating is essential because of continuous Joule heat generation inside the cable. Therefore, the HTCRC coating occupies a distinct performance regime by combining high solar reflectance, high mid-infrared emissivity, and enhanced thermal conductivity.

**Table S2**. Comparison between the HTCRC coating and representative radiative cooling materials.

| Materials | Solar reflectance | Emissivity | Thermal conductivity  (W m⁻¹ K⁻¹) |
| --- | --- | --- | --- |
| HTCRC coating (this work) | 0.92 | 0.95 | 1.28 |
| Anisotropic Cooling Aerogel [11] | 0.97 | 0.88 | 0.0169 |
| Ordered Porous PMMA [12] | 0.94 | 0.95 | 0.044 |
| PMMA/PVDF foam [13] | 0.96 | 0.97 | 0.027 |

This comparison highlights that the HTCRC coating is not intended to compete with conventional radiative cooling materials solely in terms of optical performance, but rather to address the distinct thermal-management requirement of internally heated cylindrical conductors.

**Note S8. Fabrication of nylon-reinforced HTCRC coatings**

To improve the mechanical robustness of the coating, a nylon-reinforced HTCRC structure was fabricated through a simple multilayer coating process. As shown in **Fig. S5**, a first HTCRC layer was applied onto the cable surface to form a continuous and well-adhered functional layer. After partial curing of this layer, a nylon reinforcement layer was prepared and embedded onto the coated surface to serve as a mechanical support framework. Subsequently, a second HTCRC layer was applied to fully encapsulate the nylon reinforcement and form a continuous outer surface. After final curing, this process produced a composite coating structure in which the nylon reinforcement was embedded between two HTCRC layers, thereby enhancing the mechanical robustness, crack resistance, and long-term durability of the coating while preserving the radiative cooling functionality of the outer surface.

**Figure S5.** Step-by-step fabrication process of the nylon-reinforced HTCRC coating on cable surfaces.

**Note S9. Influence of Nylon reinforcement layer on heat transport**

The Nylon reinforcement layer was introduced primarily to improve the mechanical robustness of the HTCRC coating for practical cable applications. As shown in **Fig. S6**, the Nylon layer has a thickness of approximately 0.13 mm, which is substantially smaller than the overall HTCRC coating thickness (~2 mm). In addition, the textile exhibits a porous structure that allows the HTCRC matrix to fully infiltrate the pores during fabrication. As a result, continuous heat-transfer pathways are maintained throughout the composite coating, minimizing thermal interruption at the Nylon/HTCRC interface. Experimental measurements further show that the thermal conductivity of the Nylon-enhanced HTCRC remains approximately 1.2 W m⁻¹ K⁻¹, which is essentially identical to that of the HTCRC coating without Nylon reinforcement. These results indicate that the inclusion of the porous Nylon layer has a negligible influence on the overall heat-transport performance of the coating.

**Figure S6.** Characterization of the nylon reinforcement layer and its effect on thermal transport. (a) Thickness measurement of the nylon textile (~0.13 mm). (b) SEM image showing the porous woven structure of the nylon reinforcement. (c) Thermal conductivity of HTCRC coatings with and without nylon reinforcement, demonstrating the negligible influence of the nylon layer on heat transport.

**Note S10. Dust-resistant performance under desert environments**

The primary application environment considered in this work is overhead power transmission infrastructure operating under extreme hot and arid conditions, particularly representative desert climates such as Saudi Arabia (**Figs. S7a and S7b**). In these environments, the dominant environmental contaminants affecting overhead cables are airborne dust, sand particles, and wind-driven particulate deposition. This is also evident at our local outdoor test site at KAUST near Jeddah. Another important energy infrastructure component in this environment, outdoor solar panels, shows clear dust accumulation after prolonged outdoor exposure, confirming that airborne dust and sand deposition are representative fouling sources in the studied environment (**Figs. S7c and S7d**). Therefore, the main purpose of the surface hydrophobicity in our HTCRC coating is to reduce dust adhesion, facilitate removal of loosely attached particles, and maintain stable optical performance under desert environmental exposure.

**Figure S7.** Representative environmental conditions of overhead power transmission infrastructure in Saudi Arabia. (a, b) Overhead transmission lines operating under desert conditions. (c) Outdoor photovoltaic installation at KAUST. (d) Dust accumulation observed on the surface of outdoor photovoltaic panels after environmental exposure, illustrating the prevalence of airborne dust and sand deposition in the studied environment.

The dust removal experiment was performed using representative fine desert dust particles collected from outdoor environments in Saudi Arabia. After controlled dust deposition onto the HTCRC surface, water rinsing was applied to evaluate removal behavior and recovery of the original optical appearance. The results demonstrate that the moderate hydrophobicity of the HTCRC surface effectively reduces strong particle adhesion and facilitates removal of deposited dust contaminants.

**Note S11. Abrasion resistance test**

The mechanical durability of the HTCRC coating was evaluated using a sandpaper abrasion test. The coating was fixed on a stable support, and abrasion was performed manually using silicon carbide sandpaper under hand-controlled conditions. During the test, the sandpaper was moved along the cable surface with moderate and consistent pressure to produce uniform surface wear over a defined area. The abrasion procedure was repeated for a specified number of strokes to ensure comparable treatment among samples.

Before and after the abrasion test, the optical properties of the samples were characterized to evaluate the influence of surface wear on radiative cooling performance. The solar reflectance was measured using a UV–vis–NIR spectrophotometer equipped with an integrating sphere, and the mid-infrared emissivity was determined using a Fourier transform infrared (FTIR) spectrometer. All measurements were conducted under identical conditions to ensure reliable comparison. This procedure allows assessment of the mechanical robustness of the HTCRC coating and its ability to maintain high solar reflectance and infrared emissivity after mechanical wear.

**Note S12. Accelerated UV aging test**

To evaluate the long-term UV durability of the HTCRC coating, an accelerated UV aging test was conducted at a certified third-party testing institution according to ASTM G154-23 using UVA-340 fluorescent UV lamps. The total exposure duration was 240 h, consisting of repeated cycles of 8 h UV irradiation (0.89 ± 0.02 W m⁻² nm⁻¹ at 340 nm, black panel temperature: 60 ± 3 °C) followed by 4 h condensation (black panel temperature: 50 ± 3 °C). As shown in **Figs. S8a and S8b**, no visible cracking, chalking, blistering, peeling, delamination, or other apparent degradation features were observed after the accelerated aging test. The surface morphology and appearance of the HTCRC coating remained essentially unchanged throughout the exposure period, indicating excellent UV durability and structural stability under accelerated weathering conditions.

**Figure S8**. Third-party certified accelerated UV aging test (ASTM G154) of the HTCRC coating. (a) Photograph of the UV accelerated weathering chamber used for the aging test. (b) Photographs of the HTCRC samples before and after 240 h of accelerated UV exposure, showing no visible cracking, chalking, blistering, peeling, delamination, or other obvious degradation features.

**Note S13. Salt-spray corrosion test**

To evaluate corrosion-related environmental durability, a neutral salt-spray test was conducted by a certified third-party testing institution according to ASTM B117-19 (**Figs. S9**). The test was performed using a Q-FOG CCT/1100 cyclic corrosion tester with a 5.0±1.0 wt% NaCl solution. The chamber temperature was maintained at 35±2°C, the pH of the collected solution was 6.5–7.2 at 25±2°C, and the solution collection rate was controlled at 1.5±0.5 mL per 80 cm² per hour. After 240 h of salt-spray exposure, the HTCRC coating maintained stable surface integrity without obvious cracking, peeling, delamination, or visible degradation, demonstrating promising resistance against saline and humid coastal environments relevant to the Red Sea climate.

**Figure S9.** Third-party certified salt-spray resistance test of the HTCRC coating according to ASTM B117.

**Note S14. Temperature sensor attachment in no-current outdoor experiments**

The cable temperatures in the no-current outdoor experiments were measured using K-type thermocouples attached directly to the cable surfaces. To ensure stable thermal contact, the thermocouple junctions were fixed onto the cable surface using transparent adhesive tape. The sensors were positioned on the lower side of the cables to minimize direct solar irradiation on the thermocouple junctions and reduce possible measurement artifacts caused by solar heating of the sensors. Because the cables were tested under no-current conditions, direct contact between the thermocouples and the cable surfaces did not interfere with the temperature measurement. The corresponding attachment configuration is shown in **Fig. S10**.

**Figure S10.** Thermocouple attachment configuration used for outdoor temperature measurements of insulated, bare, and HTCRC-coated cables.

**Note S15. Control experiment for distinguishing solar reflection and infrared radiative cooling**

An additional validation experiment was designed to distinguish the effects of solar reflection and infrared radiative heat dissipation (**Fig. S11**). In this experiment, three representative samples with different optical characteristics were compared under identical outdoor conditions: a conventional black insulated sample with high solar absorptivity and high infrared emissivity, a bare aluminum sample with relatively low solar absorptivity but low infrared emissivity, and the HTCRC sample with both high solar reflectance and high infrared emissivity.

**Figure S11.** Outdoor experimental demonstration of the cooling performance of HTCRC coatings. a) Photograph of the field test setup under clear-sky conditions. b) Top view of the test chamber showing insulated, bare, and HTCRC-coated samples. c) Solar reflectance and infrared emissivity of the three sample surfaces. d) Schematic diagram of the chamber setup with internal heating and temperature sensing. e) Solar irradiance and ambient temperature recorded over the three-day test period. f) Surface temperature profiles of the samples.

The experimental setup was specifically designed to minimize undesired heat exchange pathways and thereby highlight the roles of solar reflection and thermal radiation. The samples were placed in a thermally insulated chamber made of low-thermal-conductivity expanded polystyrene foam to reduce parasitic heat transfer from the surroundings. A thin polyethylene film was used as a wind shield to suppress air convection while maintaining high infrared transparency, allowing radiative heat exchange to occur. In addition, identical electric heating films were attached to the back side of each sample to simulate internal Joule heating during cable operation. This configuration enabled a more direct comparison of how different combinations of solar absorptivity and infrared emissivity affect the sample temperature under controlled thermal loading and outdoor solar exposure.

If the observed cooling effect originated only from reduced solar absorption, the bare aluminum sample would be expected to exhibit a temperature similar to that of the HTCRC sample, because both surfaces absorb substantially less solar energy than the black insulated sample. However, the experimental results show that the HTCRC sample consistently maintained a significantly lower temperature than the bare aluminum sample during daytime operation. This observation indicates that reduced solar absorption alone cannot fully explain the measured temperature difference, and that the high infrared emissivity of the HTCRC coating also plays an important role in enhancing heat dissipation and improving the overall cooling performance.

More importantly, during nighttime, when solar irradiation was absent and the contribution of solar reflection became negligible, the bare aluminum sample still exhibited a noticeably higher temperature than the HTCRC sample. Since the primary optical difference between these two samples is their infrared emissivity, this result provides direct experimental evidence that enhanced infrared radiative heat dissipation contributes significantly to the overall cooling performance. The low emissivity of the bare aluminum surface limits thermal radiation, whereas the HTCRC coating, with an infrared emissivity of approximately 0.95, can dissipate heat more effectively through thermal radiation.

Therefore, the superior cooling performance of HTCRC originates from the synergistic combination of suppressed solar heat gain and enhanced infrared radiative heat dissipation. High solar reflectance minimizes solar heat absorption during daytime operation, while high infrared emissivity facilitates thermal radiation from the heated surface. Together, these effects enable substantially lower operating temperatures than can be achieved by solar reflection alone.

**Note S16. Demonstration of sub-ambient radiative cooling under non-energized conditions**

An additional passive radiative cooling experiment was performed to evaluate the intrinsic radiative cooling capability of the HTCRC coating under non-energized conditions (**Figs. S11a and S11b**). The experimental design followed the recommendations outlined in *Best Practices for Radiative Cooling* (Nat. Sustain. 6 2023 1030-1032) to minimize parasitic heat transfer and ensure reliable assessment of radiative cooling performance. In the experiment, the HTCRC sample was placed inside a chamber constructed from expanded polystyrene (EPS) foam with extremely low thermal conductivity to suppress conductive heat exchange with the surroundings. A thin polyethylene film, which is highly transparent in the mid-infrared atmospheric window, was used as a wind shield to minimize convective heat transfer while preserving radiative exchange with the sky. Unlike the heating experiments discussed in **Note S15**, no electrical heating was applied in this test, allowing the intrinsic passive cooling behavior of the coating to be directly evaluated.

The experimental results demonstrate that the HTCRC coating achieved clear sub-ambient cooling under outdoor daytime conditions. As shown in **Fig. S12**, the surface temperature of the HTCRC sample remained below the ambient air temperature throughout the daytime period and reached a maximum temperature reduction of approximately 6°C below ambient temperature near solar noon. This result confirms that the coating is capable of rejecting more heat than it absorbs from solar irradiation and the surrounding environment under realistic outdoor conditions. To further quantify the cooling performance, the net radiative cooling power was calculated based on the measured optical properties of the HTCRC coating and the corresponding environmental conditions during the experiment. The calculated net cooling power reached approximately 84 W m⁻², indicating a positive net radiative heat flux from the coating to the sky.

**Figure S12.** Daytime passive radiative cooling performance of the HTCRC coating under non-energized outdoor conditions.

Therefore, the additional experiment satisfies the two key criteria commonly adopted for rigorous passive radiative cooling characterization: (i) positive net radiative cooling power and (ii) sub-ambient cooling under non-energized conditions. These results confirm that the HTCRC coating functions not only as a high-emissivity heat-dissipation layer for cable thermal management, but also as a genuine radiative cooling material capable of achieving passive cooling below ambient temperature.

**Note S17. Simulated cables**

**Figure S13a** illustrates the cross-sectional structure of the HTCRC-coated simulated cable used in this study. The simulated cable was designed to reproduce the thermal behavior of an overhead conductor under controlled laboratory conditions. The core consists of a nichrome resistance alloy wire placed inside a high-temperature-resistant silicone tube, which provides electrical insulation and thermal stability during heating. Owing to its relatively high electrical resistivity (~2 Ω m⁻^1^) the nichrome wire can generate sufficient heat under a low operating current (~5 A), enabling the cable to reproduce the Joule heating effect of real overhead aluminum conductors while ensuring safe and stable operation in laboratory environments. This configuration allows the temperature rise, heat dissipation, and radiative cooling performance of the coated cable to be evaluated under well-controlled conditions without the need for high-current power supplies.

The outer surface of the simulated cable is coated with the HTCRC layer (**Figure S13b**), enabling direct comparison of temperature evolution and thermal performance between coated and uncoated configurations. Thermocouples are attached to the cable surface to monitor temperature in real time, allowing quantitative assessment of the cooling effect and the corresponding implications for conductor thermal management.

**Figure S13.** Design and outdoor demonstration setup of simulated cables. (a) Schematic of the cable cross section showing the internal heating structure and surface thermocouple placement. Joule heat is generated by nichrome-resistance wires embedded within a silicone tube. (b) Photograph of three simulated cables with insulated, bare, and HTCRC-coated surfaces under natural solar exposure.

**Note S18. Meteorological conditions during outdoor cable measurements**

The meteorological conditions corresponding to the outdoor cable cooling experiment shown in **Fig. 4e** were recorded simultaneously using a weather station. As shown in **Figure S14**, the measured parameters include ambient temperature, solar irradiance, wind speed, and relative humidity over the seven-day measurement period from August 2 to August 8, 2025. These data provide the environmental context for interpreting the measured cable temperatures under realistic outdoor operating conditions.

**Figure S14.** Temporal variations of the meteorological conditions during the outdoor experiments. (a) Ambient temperature, (b) solar irradiance, (c) wind speed, and (d) ambient relative humidity.

**Note S19 Overhead conductor sag model**

Reducing the sag of overhead conductors is one of the important functions of our radiative cooling coating. Considering that it is not feasible to perform full scale experiments on actual conductors to directly demonstrate the sag reduction, we employ the catenary formulation to quantitatively evaluate the sag. The catenary equation describes the equilibrium profile of a suspended conductor under its own weight and is widely used to determine the lowest point of the conductor, the span wise shape, the mechanical tension, and the safety clearance under varying meteorological conditions. In the absence of significant wind load or ice accretion, the exact shape of the conductor subjected only to self-weight can be expressed as:

$$y\left(s\right)=a\cosh\left(\frac{x}{a}\right)-a$$

where $y\left(x\right)$ is the vertical displacement from the lowest point, *x* is the horizontal coordinate, and $a=H/w$ is the catenary constant, with *H* representing the horizontal component of the conductor tension and *w* the conductor’s unit weight (N m⁻^1^). The mid-span sag *D* can then be calculated as:

$$D=a\left[\cosh\left(\frac{L}{2a}\right)-1\right]$$

where *L* is the span length. To account for temperature effects and long-term deformation, the catenary geometry is coupled with a thermal-mechanical-creep length compatibility relation. Let $S_{0}$ be the conductor length within the span at a reference temperature $T_{0}$ and a reference horizontal tension $H_{0}$. At temperature *T* and time *t*, the span length becomes. The conductor length at temperature *T* differs from a reference length $S_{0}$ at temperature $T_{0}$ due to both thermal expansion and elastic elongation, expressed as:

$$S\left(T,\text{ }t\right)=S_{0}\left[1+\alpha\left(T-T_{0}\right)+\frac{H\left(T,\text{ }t\right)-H_{0}}{E_{\text{c}}A}\right]+S_{\text{creep}}\left(t\right)$$

where $\alpha$ is the coefficient of thermal expansion, $E_{\text{c}}$ is the effective elastic modulus, and *A* is the cross-sectional area of the conductor, and $S_{\text{creep}}\left(t\right)$ denotes the permanent elongation accumulated over time due to creep under service stress and temperature. In practical conductor sag calculations, the cross-sectional area is assumed constant, as the axial strain-induced area change is typically less than 0.1% even for large temperature variations, and thus has negligible influence on mechanical tension and sag predictions. The mechanical compatibility condition equates this length to the geometrical length given by the catenary equation:

$$S\left(T,\text{ }t\right)=2a\left(T,\text{ }t\right)\sinh\left(\frac{L}{2a\left(T,\text{ }t\right)}\right),\text{ }a\left(T,\text{ }t\right)=\frac{H\left(T,\text{ }t\right)}{w}$$

By solving this coupled system iteratively for $H\left(T,\text{ }t\right)$, the temperature-dependent sag $D\left(T,\text{ }t\right)$ can be obtained directly from the catenary relation. This framework enables quantitative prediction of sag reduction as a direct result of temperature decreases achieved by radiative cooling coatings, providing a clear link between thermal management performance and mechanical reliability of overhead conductors.

The term $S_{\text{creep}}\left(t\right)$ represents time dependent irrecoverable strain. In practice it can be introduced through an empirical law calibrated from material or conductor level tests. A common representation uses a logarithmic form

$$\varepsilon_{\text{creep}}\left(t\right)=\frac{S_{\text{creep}}\left(t\right)}{S_{0}}=K\left(\sigma,\text{ }T\right)\log_{10}\left(\frac{t}{t_{\text{ref}}}\right)$$

where$K\left(\sigma,\text{ }T\right)$ depends on the operating stress$\sigma=H/A$and temperature *T*, and $t_{\text{ref}}$ is a reference time. If desired, the temperature sensitivity can be captured by writing

$$K\left(\sigma,\text{ }T\right)=K_{0}\left(\sigma\right)\exp\left(-\frac{Q}{RT}\right)$$

with an apparent activation energy *Q* and the gas constant *R*. Once $S_{\text{creep}}\left(t\right)$ is specified, the solution proceeds by iteratively finding $H\left(T,\text{ }t\right)$ that satisfies

$$r\left(H;T,t\right)=\left[2\frac{H}{\omega}\sinh\left(\frac{L\omega}{2H}\right)-2\frac{H_{0}}{\omega}\sinh\left(\frac{L\omega}{2H_{0}}\right)\right]-S_{0}\left[\alpha\left(T-T_{0}\right)+\frac{H-H_{0}}{E_{\text{c}}A}\right]-S_{\text{creep}}\left(t\right)=0$$

followed by

$$D\left(T,\text{ }t\right)=\alpha\left(T,\text{ }t\right)\left[\cosh\left(\frac{L}{2\alpha\left(T,\text{ }t\right)}\right)-1\right].$$

In the short-term limit where $S_{\text{creep}}\left(t\right)$ is negligible, the formulation reduces to the purely thermo mechanical case used earlier. In long term assessments, the creep term increases the effective span length, reduces the horizontal tension, and therefore increases sag even at constant temperature. This framework directly links temperature reduction achieved by radiative cooling with a lower creep rate and a smaller long term sag growth, which is essential for evaluating the reliability benefit of the coating.

**Note S20. Thermal and ampacity model**

According to CIGRE TB 601 [14], under steady-state conditions, where the weather parameters, conductor current, and conductor temperature remain constant, the thermal equilibrium of an overhead conductor under actual environmental conditions can be described by a power balance equation. In this formulation, the left-hand side represents the heat gain terms (W m⁻^1^) that contribute to conductor heating, whereas the right-hand side represents the heat loss terms (W m⁻^1^) associated with conductor cooling:

$$P_{\text{j}}+P_{\text{s}}=P_{\text{c}}+P_{\text{r}}$$

where *P*_j_ is the heating of the conductor by the current flowing (Joule heating), *P*_s_ is the heating of the conductor by the sunlight (solar heating), *P*_c_ is the cooling of the conductor by the convection (convective cooling, natural and forced convection), *P*_r_ is the cooling of the conductor by the radiation (radiative cooling).

For heavily loaded transmission lines, Joule heating plays a dominant role in determining the current-carrying capacity of the conductor. This heat source originates from the electrical energy dissipated as current flows through the conductor and depends on both the DC resistance and the additional resistance associated with the skin effect under alternating current conditions. The linear heat generation rate due to Joule heating, *P*_j_ (W m⁻^1^), can be expressed as:

$$P_{\text{j}}=I_{\text{AC}}^{2}R_{\text{AC }20}\left[1+\alpha\left(T_{\text{s}}-20\right)\right]$$

where *I*_AC_ denotes the root-mean-square (RMS) value of the alternating current (A), *R*_AC 20_ is the AC resistance of the conductor at 20 °C including skin-effect contributions (Ω m⁻^1^), *α* is the temperature coefficient of resistance (K^−1^ or °C ^−1^), and *T*_s_ represents the conductor temperature (°C). In addition to electrical heating, solar radiation contributes to the thermal load of overhead conductors. The solar heat gain *P*_s_ (W m⁻^1^) can be written as:

$$P_{\text{s}}=\alpha_{\text{s}}SD$$

where $\alpha_{\text{s}}$ is the solar absorptivity of the conductor surface (dimensionless), *S* is the incident solar irradiance (W m⁻^2^), and *D* is the outer diameter of the conductor (m). The convective heat loss *P*_c_ (W m⁻^1^) is given by:

$$P_{\text{c}}=\pi\lambda_{\text{f}}\left(T_{\text{s}}-T_{a}\right)Nu$$

where *Nu* is the Nusselt number. Radiative heat transfer represents another important cooling mechanism, corresponding to the net thermal radiation emitted from the conductor surface to the surroundings and the sky. According to the Stefan–Boltzmann law, the radiative heat loss *P*_r_ (W m⁻^1^) can be expressed as:

$$P_{\text{r}}=\pi D\varepsilon_{\text{s}}\sigma_{\text{B}}\left[\left(T_{\text{s}}+273\right)^{4}-\left(T_{\text{a}}+273\right)^{4}\right]$$

where $\varepsilon_{\text{s}}$ is the surface emissivity of the conductor (dimensionless) and $\sigma_{\text{B}}$ is the Stefan–Boltzmann constant, $\sigma_{\text{B}}$= 5.6697·10^−8^ W m⁻^2^ K⁻^4^. Based on the overall thermal balance between heat gain and heat dissipation, the maximum allowable current (ampacity, $I_{\max}$) corresponding to a conductor temperature not exceeding *T*_s_ can be determined:

$$I_{\max}=\sqrt{\frac{P_{\text{c}}+P_{\text{r}}-P_{\text{s}}}{R_{\text{AC }20}\left[1+\alpha\left(T_{\text{s}}-20\right)\right]}}$$

**Note S21. Meteorological inputs used in the ampacity analysis**

To clarify the environmental conditions considered in the ampacity calculations, location-dependent meteorological data were incorporated into the CIGRE 601 thermal rating model. The meteorological inputs, including ambient temperature, solar irradiance, wind speed, and wind direction, were obtained from the commercial weather database platform Visual Crossing Weather Data, which provides historical climate and weather information for different geographic locations worldwide. These data were used for the selected global cities and seasonal operating conditions considered in the ampacity analysis. As a representative example, **Fig. S15** shows the meteorological inputs for one summer day in Riyadh used in the extreme-summer ampacity calculation. Rather than assuming constant environmental conditions, the model incorporates time-varying meteorological data throughout the simulation period. Therefore, the reported ampacity enhancement reflects the combined influence of realistic environmental fluctuations and the improved thermal-management capability provided by the HTCRC coating. For example, during the representative summer day in Riyadh, the ambient temperature exceeds 40 °C while the solar irradiance approaches 1000 W m⁻² during midday. Under these conditions, the HTCRC coating provides the largest ampacity enhancement owing to its ability to simultaneously suppress solar heat gain and enhance heat dissipation. These meteorological conditions form the basis of the extreme-summer ampacity results presented in **Fig. 5** in the manuscript.

**Figure S15.** Representative meteorological inputs used for the ampacity calculation based on one day in July in Riyadh. (a) Ambient temperature, (b) solar irradiance, (c) wind speed, and (d) wind direction.

**Note S22. Preliminary cost analysis of the HTCRC coating**

The proposed HTCRC coating is composed of commercially available PDMS and industrial-grade h-BN fillers. Based on representative industrial raw-material prices and the coating formulation used in this work, the estimated material cost of the HTCRC coating is approximately $10 m⁻². For comparison, commercial passive radiative cooling products represented by SPACECOOL, i2Cool, and 3M are mainly designed for building energy-saving applications, with reported coating costs averaging approximately $6.29 m⁻² [15].

Although the incorporation of high-thermal-conductivity h-BN fillers makes the HTCRC coating moderately more expensive than conventional radiative cooling coatings based on low-cost inorganic fillers, this additional cost corresponds to its application-specific functionality. Unlike conventional radiative cooling coatings primarily designed to suppress external solar heat gain on static planar surfaces, the HTCRC coating targets internally heated cylindrical conductors, where efficient heat extraction from the cable core is essential because of continuous Joule heat generation. Therefore, high thermal conductivity is required to rapidly transport internally generated heat to the coating surface for subsequent convective and radiative dissipation.

Considering the potential benefits of reduced Joule losses, enhanced ampacity, mitigated thermal sag, and improved grid reliability, the practical value of the HTCRC coating should be evaluated at the system level rather than solely by raw material cost. Future cost reduction may be achieved by using industrial-grade h-BN fillers, optimizing filler loading, introducing hybrid filler systems, or designing multilayer coating architectures with selective placement of high-conductivity fillers.

**References**

[1] F. Wang, H. Wang, D. Gong, Z. Cheng, L. Ma, Radiative transfer analysis of semitransparent medium with particles having non-uniform size distribution by differential-integration method, **Int. J. Heat Mass Transf.** 130 (**2019**) 342-355.

[2] C. F. Bohren, D. R. Huffman, Absorption and scattering of light by small particles, John Wiley & Sons, 2008.

[3] M. Brewster, C. Tien, Radiative transfer in packed fluidized beds: dependent versus independent scattering, **J. Heat Transfer** 104 (**1982**) 573-579.

[4] J. Peoples, X. Li, Y. Lv, J. Qiu, Z. Huang, X. Ruan, A strategy of hierarchical particle sizes in nanoparticle composite for enhancing solar reflection, **Int. J. Heat Mass Transfer** 131 (**2019**) 487-494.

[5] D. Duncan, The colour of pigment mixtures, **Proc. Phys. Soc.** 52 (**1940**) 390.

[6] L. Wang, S. L. Jacques, L. Zheng, MCML—Monte Carlo modeling of light transport in multi-layered tissues, **Comput. Methods Programs Biomed.** 47 (**1995**) 131-146.

[7] D. B. Judd, Fresnel reflection of diffusely incident light, **J. Res. Natl. Bur. Stand.** 29 (**1942**) 329-332.

[8] B. Hunter, Z. Guo, Conservation of asymmetry factor in phase function discretization for radiative transfer analysis in anisotropic scattering media, **Int. J. Heat Mass Transfer** 55 (**2012**) 1544-1552.

[9] H. Pradlwarter, G. Schuëller, On advanced Monte Carlo simulation procedures in stochastic structural dynamics, **Int. J. Non-Linear Mech.** 32 (**1997**) 735-744.

[10] P. Chýlek, G. Grams, R. Pinnick, Light scattering by irregular randomly oriented particles, **Science** 193 (**1976**) 480-482.

[11] K.-Y. Chan, X. Shen, J. Yang, K.-T. Lin, H. Venkatesan, E. Kim, H. Zhang, J.-H. Lee, J. Yu, J. Yang, Scalable anisotropic cooling aerogels by additive freeze-casting, **Nature Communications** 13 (**2022**) 5553.

[12] Y. Zhang, T. Wang, X. Mei, M. Chen, L. Wu, Ordered porous polymer films for highly efficient passive daytime radiative cooling, **ACS Photonics** 10 (**2023**) 3124-3132.

[13] W. Zhong, Y. Chen, D. Hu, J. Sun, X. Jia, L. Zhao, Unprecedented Ultra‐High Expansion Ratio Foam for Innovative Architecture, **Advanced Science** 12 (**2025**) 2501188.

[14] M. Kanálik, A. Margitová, Ľ. Beňa, Temperature calculation of overhead power line conductors based on CIGRE Technical Brochure 601 in Slovakia, **Electrical Engineering** 101 (**2019**) 921-933.

[15] https://[www.intelmarketresearch.com/passive-daytime-radiative-cooling-materials-market-market-3781](http://www.intelmarketresearch.com/passive-daytime-radiative-cooling-materials-market-market-3781).
